# Additive manufacturing of titanium-based alloys- A review of methods, properties, challenges, and prospects

**DOI:** 10.1016/j.heliyon.2022.e09041

**Published:** 2022-03-07

**Authors:** Thato Sharon Tshephe, Samuel Olukayode Akinwamide, Eugene Olevsky, Peter Apata Olubambi

**Affiliations:** a Centre for Nanomechanics and Tribocorrosion, School of Mining, Metallurgy and Chemical Engineering, University of Johannesburg, South Africa; b Department of Mechanical Engineering, San Diego State University, USA

**Keywords:** Additive manufacturing, Titanium alloy, Aerospace, Biomedical, Automobiles, Corrosion, Mechanical properties

## Abstract

The development of materials for biomedical, aerospace, and automobile industries has been a significant area of research in recent years. Various metallic materials, including steels, cast iron, nickel-based alloys, and other metals with exceptional mechanical properties, have been reportedly utilized for fabrication in these industries. However, titanium and its alloys have proven to be outstanding due to their enhanced properties. The β-titanium alloys with reduced modulus compared with the human bone have found more usage in the biomedical industry. In contrast, the α and α+β titanium alloys are more utilized to fabricate parts in the automobile and aerospace industries due to their relatively lightweight. Amongst the numerous additive manufacturing (AM) techniques, selective laser and electron beam melting techniques are frequently used for the fabrication of metallic components due to the full densification and high dimensional accuracy they offer. This paper reviews and discusses the different types of AM techniques, attention is also drawn to the properties and challenges associated with additively manufactured titanium -based alloys. The outcome from this study shows that 3D printed titanium and titanium-alloys exhibit huge prospects for various applications in the medical and aerospace industries. Also, laser-assisted 3D technologies were found to be the most effective AM method for achieving enhanced or near-full densification.

## 1 Introduction

AM technique is a recently developed method, reportedly used to fabricate parts in industries, including biomedical, aerospace, and automobile [1, 2, 3]. This method invented by Chucks Hull in the 1980s uses 3D digital design data in building different component layers through material deposition [4, 5]. The building of intricate shapes accompanied by complex geometry and design, which are difficult to produce using other conventional methods such as casting [6], and powder metallurgy [7], are easily fabricated at higher speed and accuracy when AM processing route is adopted [8].

AM is commonly used due to several factors, including fast prototyping, complex geometry, fabrication of several combined parts, enhanced performance, and low volume manufacturing [9]. This method that is still gaining momentum has gained attention in the last decade owing to several benefits such as improved automation, wide availability of CAM/CAD design software, and a growing library of printable materials [5]. This fabricating technique requires an in-depth knowledge of the microstructure and mechanical properties resulting from manufactured components. However, it is noteworthy that the resulting properties will depend greatly on the AM route adopted for fabrication.

The frequently used AM methods include vat photopolymerization which cures materials by light-activated polymerization, material jetting in which droplets of materials are jetted to join powder materials, powder bed fusion where energy is used for selective fusion of powder bed regions and material extrusion where materials are dispersed through a nozzle before solidification [10, 11]. In addition, an extensive investigation has been carried out on the fabrication of unalloyed titanium and other alloys of titanium using AM techniques [12, 13].

Recently, titanium metal and its alloys have been a material of interest in the biomedical [14, 15], aerospace [16, 17], defense [18, 19], and automobile industries [20, 21, 22]. This has been attributed to the exceptional improved strength, wear, biocompatibility, excellent corrosion resistance, and low modulus of elasticity they possess. The titanium alloy grades presented in Table 1 have been reportedly used in several engineering industries. However, research has shown that the alloys' overall properties depend on the degree of impurity they contained, which adversely affects their plasticity [23, 24, 25]. Titanium alloys undergo plastic deformation when hydrogen, carbon, oxygen, and nitrogen are present [26, 27]. In general, alloying elements play a crucial role in stabilizing its α or β phase [28, 29]. The incorporation of the alloy elements in varying proportions often results in the formation of phases which include near- α, α near-β, α-β, β -titanium and metastable β titanium alloys [30, 31].

**Table 1:** Application and mechanical properties of different grades of titanium based alloys.

Titanium alloy	Industry	Tensile strength (MPa)	Elastic modulus (GPa)	Application	Ref
Ti–8Al–1Mo–1V	Aerospace	897	117	Compressor blades, Hydraulic lifts	[44, 45]
Ti–10V–2Fe–3Al	✓	970	900	High strength airframe components	[46, 47, 48]
Ti–13Al–11Cr–3Al	✓	1276	101.4	Wire springs	[49]
Ti–6Al–2Sn–4Zr–6Mo	✓	1210	114	Hydraulic systems	[50, 51]
Ti–Al-2.5	✓	620	100	Aircraft engine	[52]
Ti–6Al–4V	Automobile	117	897	Connecting rods, wheel rim screws	[20, 53, 54]
Ti grade 1s	✓	105–120	345	Brake seal washers, valves	[55, 56, 57]
Timetal LCB	✓	-	1565	Suspension springs	[57, 58]
Β-titanium alloys	✓	101.4	1300	Valve springs	[59, 60]
Ti–12Mo–6Zr–2Fe	Biomedical	74	585	Hip replacement	[61, 62]
Ti–35Nb–7Zr–5Ta	✓	55	500	Dental implant	[63, 64, 65]
Ti–29Nb–13Ta-4.6Zr	✓	59	650	Artificial knee joint	[66, 67, 68]
Ti–35Nb–7Zr–5Ta	✓	55	550	Orthopedic implant	[69]
Ti–29Nb–13Ta-4.6Zr	✓	59	650	Cortical bone	[70]
Ti–24Nb–4Zr–8Sn	✓	42	850	Spine joints and bone plates	[71]

**Figure 1:**
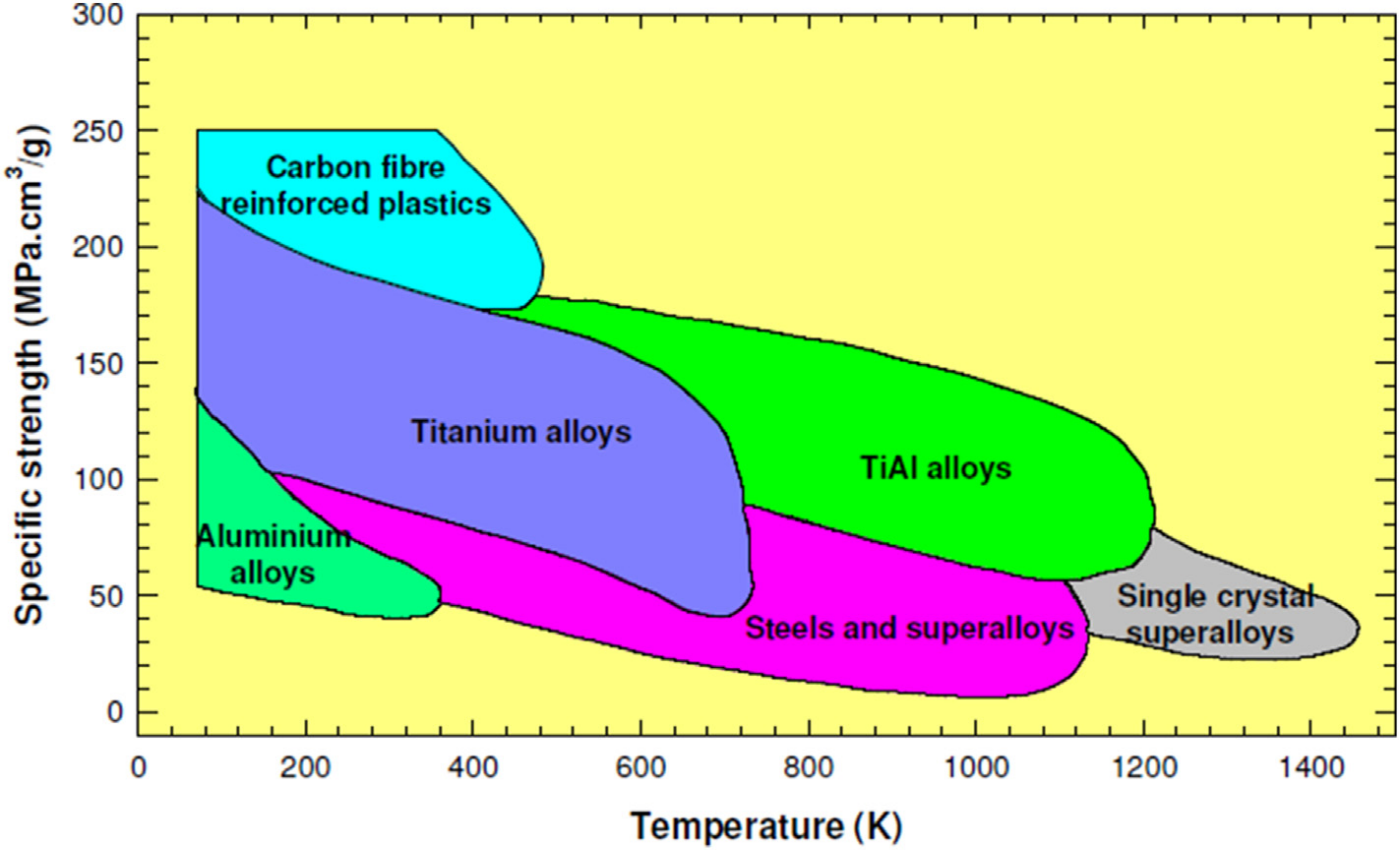
A plot of specific strength of titanium alloys against the thermal strength of other structural materials [36].

Titanium aluminide (TiAl) alloys have generated significant interests in several applications, predominantly in structural systems such as aerospace and automobiles, where lightweight and high strength/weight ratios are required [32]. Titanium aluminide alloy has high-temperature strength and improved oxidation resistance (>750 °C), and this makes them fit for high-temperature structural applications [33]. Other types of titanium alloys such as alpha type titanium alloys, dual phase titanium alloys, metastable and stable beta titanium alloys have also been employed in various industrial sectors [34]. A comparison between the strength exhibited by titanium alloys at elevated temperatures and other metallic alloys is illustrated in Figure 1. The replacement of parts produced from other metallic-based superalloys with titanium in aerospace applications is expected to decrease the structural weight of gas turbine engines with high performance by approximately 30% [35]. Therefore, a slight increase in fuel efficiency and performance may be noticeable when titanium alloys are utilized to manufacture engine parts for gas turbines.

Furthermore, other conventional techniques such as powder processing, forging, casting, and powder metallurgy processes have been reportedly used for the production of Ti-based alloys [37, 38, 39, 40]. However, these techniques often pose some challenges during the processing, leading to increased production costs [41].

AM has been reported as a promising technology with extensive design freedom in geometry [42, 43]; therefore, immense attention has been geared towards finding a suitable AM process to fabricate titanium alloy parts with improved mechanical properties. This review will provide a general overview of AM fabrication techniques and provide information on microstructural and mechanical characterization of titanium-based alloys fabricated via various AM methods.

## 2 Reported AM processes utilized for fabrication of titanium-based alloys

Different AM methods has attracted global research interest over the past few years owing to its exceptional benefits over other traditional manufacturing processes. AM enables the fabrication of physical components from computer-aided design (CAD) files in a 3D printing machine by joining materials in layers [72, 73]. Moreover, this new technology brings about many innovations such as shortened product development cycle, fabrication of complex parts, which are difficult to fabricate using other conventional production technique; energy, materials, and human resources, can be appreciably reduced. AM methods are essentially classified by the properties of the feedstock and binding mechanism between the material layers joined together [74]. Several investigations have been carried out on alloying of titanium alloys with elements such as Cr, Mo, Nb, V, Si using AM technologies for different biomedical and aerospace industries. However, there are very few successful attempts to fabricate titanium alloys using binder jetting processes to produce a 3D component with improved mechanical properties as the process operates at room temperature and do not have adequate temperature to fuse powders together for an enhanced green density.

### 2.1 Electron beam melting (EBM) process

**Figure 2:**
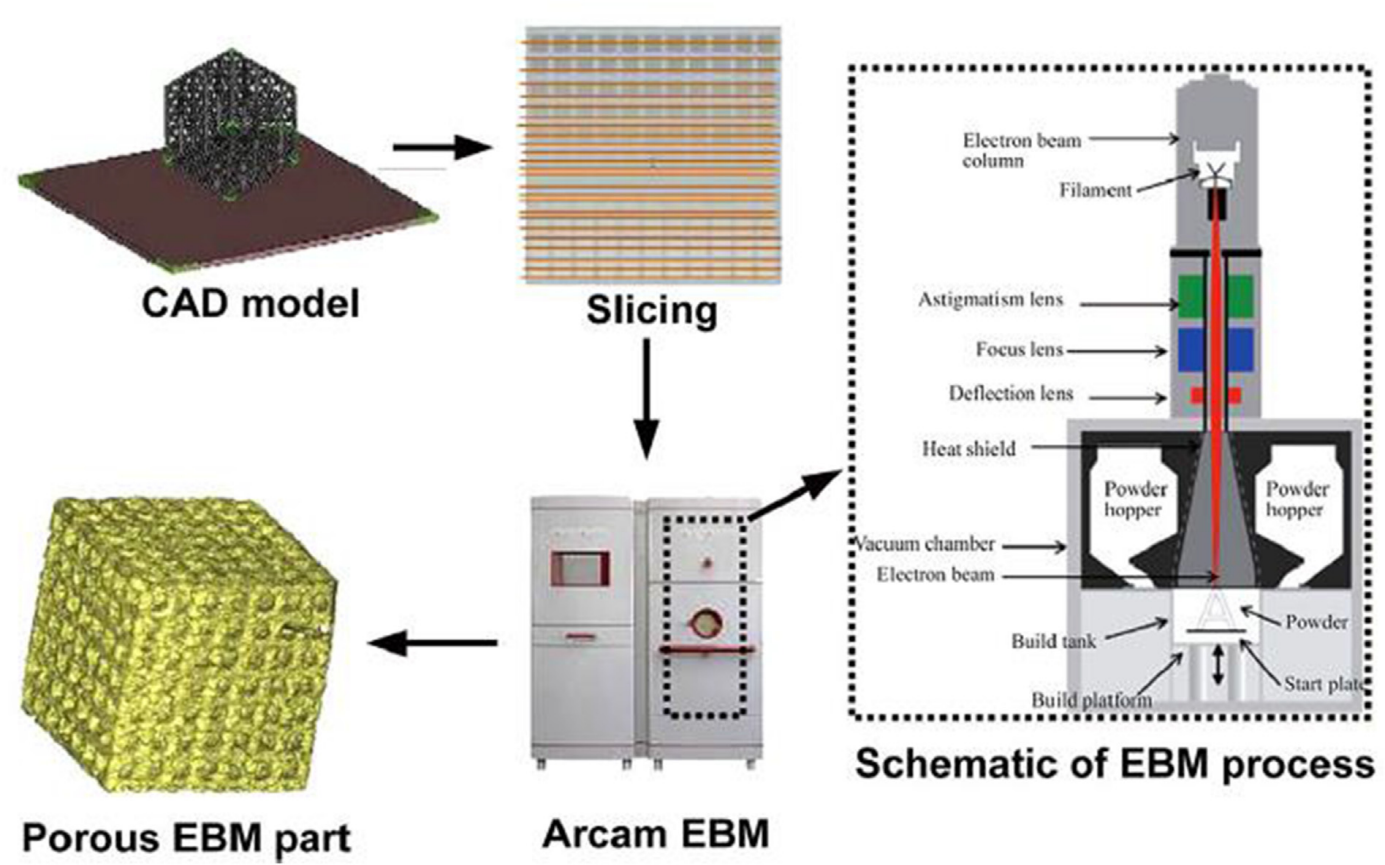
Schematic illustration of electron beam melting system [84].

EBM has recently attracted attention in manufacturing industries due to its ease of fabrication of defect-free metallic components. This AM technique represented in Figure 2 adopts an electron gun controlled by a computer to create fully dense 3D components from metallic powders [75, 76]. Structural components are generated through selective melting of electrically conductive powders by electron beams under a controlled vacuum. It should also be noted that parameters such as powder conductivity and sintering temperature adopted for fabrication are fixed, and this helps determine the boundary condition of preheating temperature [76]. In contrast, other processing parameters such as electron beam scan rate, scanning strategy, and scanning rate should be carefully calculated and optimized [77]. EBM has been successfully used to fabricate parts in the automotive, aerospace, and medical implant industries [78, 79, 80]. This makes the fabricated components strong, void-free, and fully dense. Biamano et al. [81] explored the possibility of producing near-net-shape components from Ti–48Al–2Cr–2Nb (45 and 150 μm) alloy composition. Process optimization was needed to eliminate the residual porosity located between two subsequent layers. This study proved EBM to be an ideal AM technique if the fabrication of titanium-based alloys with little internal defect and homogeneously dispersed alloying elements is desired.

Rastkar and Shokri [82] reported on the phase evolution of Ti–45Al–2Nb–2Mn–1B gamma-based titanium aluminide fabricated via EBM technique. Results from this study showed that the lamellar structure of the fabricated alloy, which was initially composed of α_2_ (Ti3Al) dendrites, became transformed into an interdendritic γ (TiAl) phase. Moreover, this phase transformation increased the surface hardness to 600 HV in comparison to a value of 330 HV recorded for the untreated specimen. A further observation from this study showed few cracks on the surface layers at lower energies and higher speeds. In comparison, lower speeds and higher energies produced hard crack resistant surface layers.

The optical micrograph of the produced specimen's vertical section revealed a microstructure which consist of fine equiaxed and little coarse grains. The fine gamma equiaxed microstructure was attributed to rapid cooling. Furthermore, series of heat treatments were performed on the specimens to obtain a near lamellar microstructure, which is a major requirement in the automotive industry [83]. The microstructure after heat treatment consisted of both equiaxed fine and coarse grains. It should be noted that the microstructure can be easily transformed upon subsequent heat.

### 2.2 Directed energy deposition (DED) process

The DED technique adopts a laser beam to deposit a pool of molten metal to a substrate, in which metallic powder is injected through a gas stream [85, 86]. This method is suitable for the fabrication of metallic components, including screws, valves, and mold tools [87, 88]. DED is currently replacing several other conventional manufacturing processes such as thermal spraying and gas metal arc welding. Recently, Liu et al. [89] published their findings on the ductility property of heat treated Ti–5Al–5Mo–5V–1Cr–1Fe titanium alloy, fabricated using DED technique. To prevent the oxidation of the melt pool, the experiments were reportedly conducted inside a chamber purged with argon. The dimensions of the fabricated plate-like sample were approximately 400 mm × 300 mm × 40mm. Annealing heat-treatment was carried out at 750 °C for 2 h and then cooled in air. Microstructural examination of the samples reportedly showed that they exhibit an ultrafine basket-weave structure, with high strength but reduced ductility. After The authors pointed out that the specimens showed an improved ductility with a decreased strength after annealing. However, this behaviour was ascribed to continuous formation of αGB, which resulted in α phase precipitate-free zones. Thomas et al. [90] examined the laser metal deposition of Ti–47Al–2Cr–2Nb alloy. The scan rate was between 60 and 4000 mm/min during the first trial for different laser power and different powder feed rate. The main feature recorded in the single bead was the presence of microcracks under a microscope, formed in the transverse direction.

**Figure 3:**
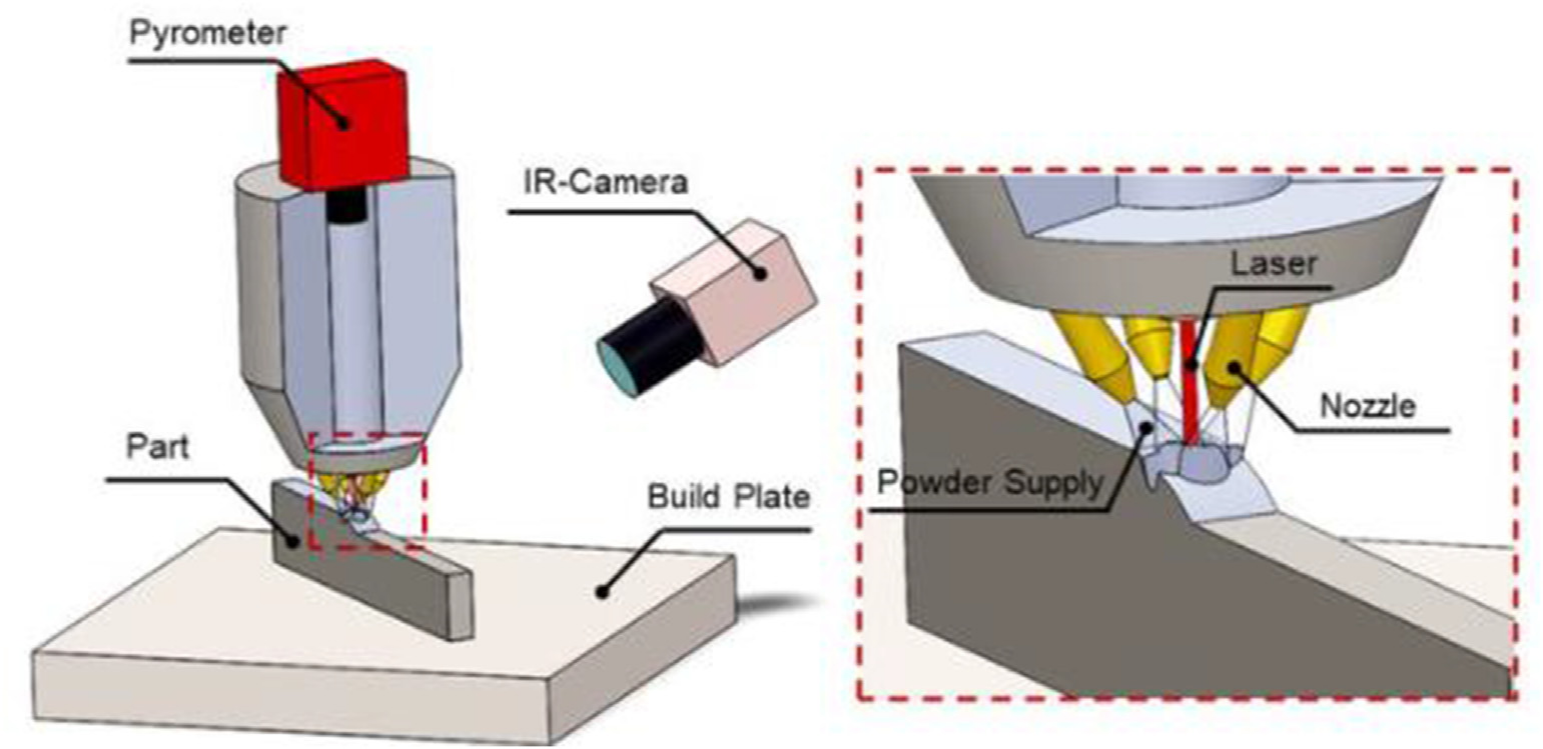
A schematic illustration of the directed energy deposited [91].

Overall, these studies described DED as a helpful technique for manufacturing and repairing complex parts in an automated fashion due to its low heat input, which helps the material retain its strength. It also produces a metallurgical bond between the deposited metal and base material, promoting adhesion between parts. A schematic representation of this process is presented in Figure 3.

### 2.3 Binder jetting printer (BJP) process

**Figure 4:**
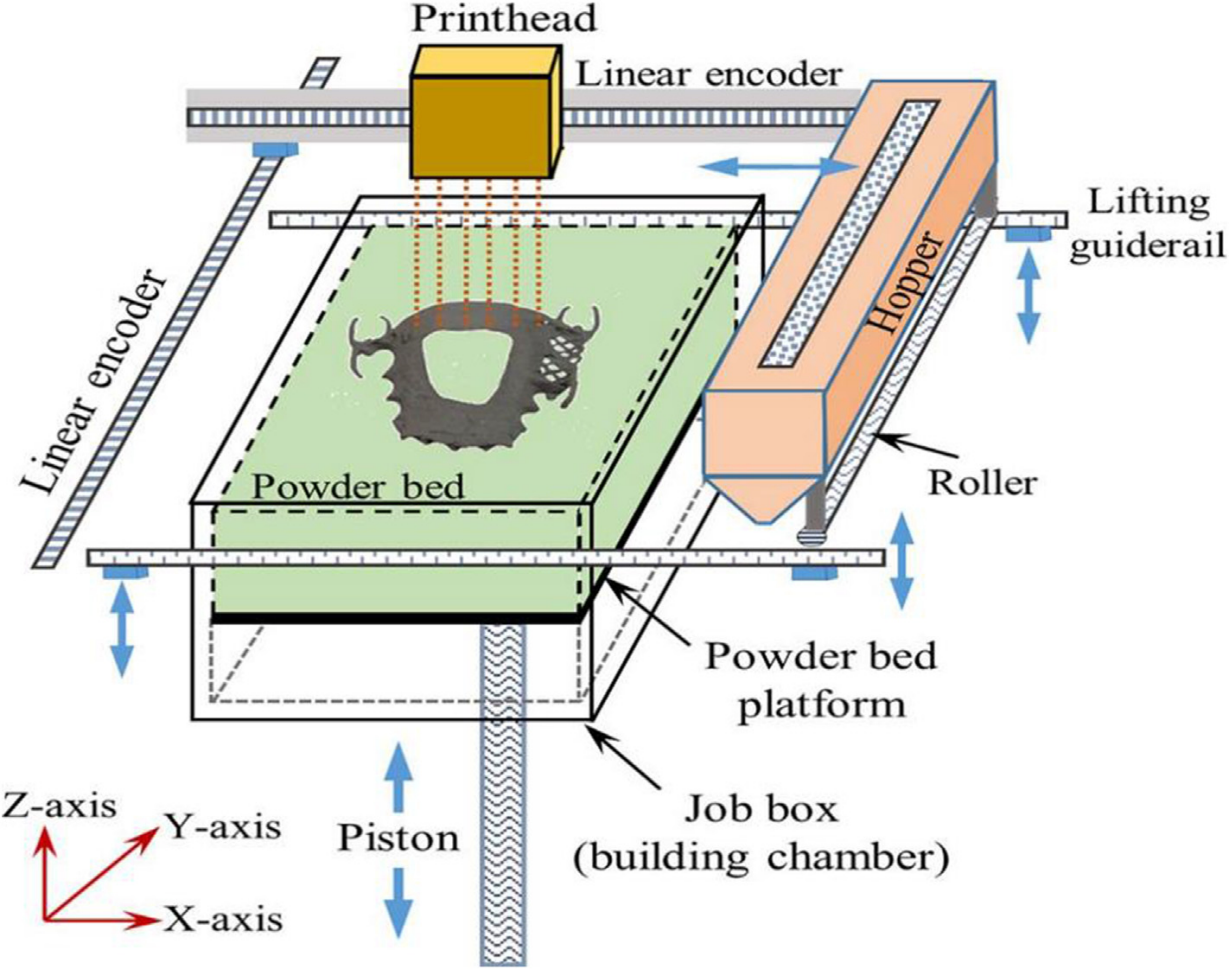
Binder jet printing system [93].

Binder jetting, also referred to as binder jet printing, is a technique in which metal powder is deposited in layers and joined selectively with a polymeric liquid binder. A diagram of a binder jet process is presented in Figure 4. The strength of binder jetted parts can be increased by fully curing the binder, and then unbound powder is removed. The metal powder is then sintered for densification or infiltrated with a metal which has a reduced melting point metal [92].

**Figure 5:**
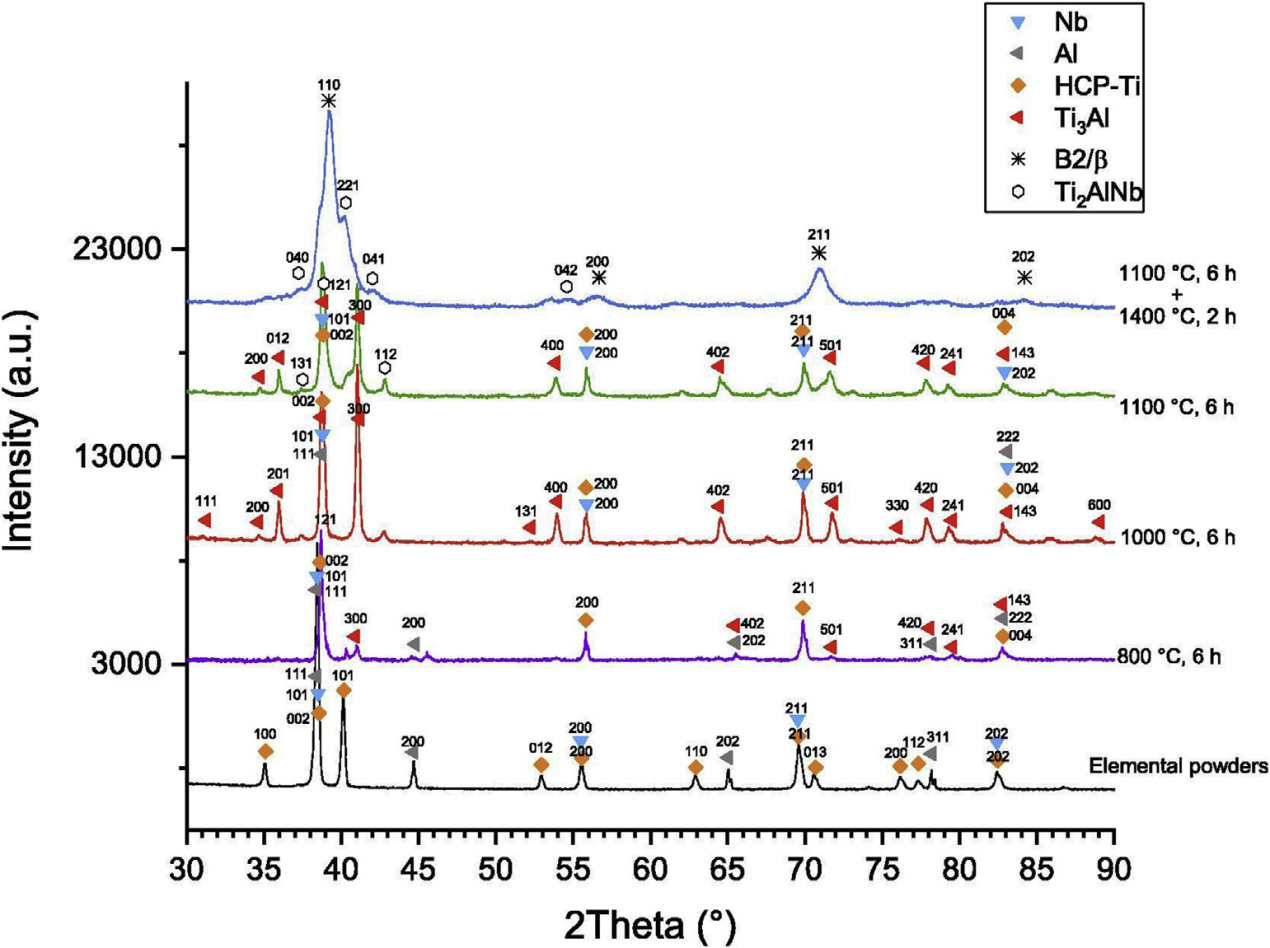
XRD plots of mixed powders and post treated samples [94].

**Figure 6:**
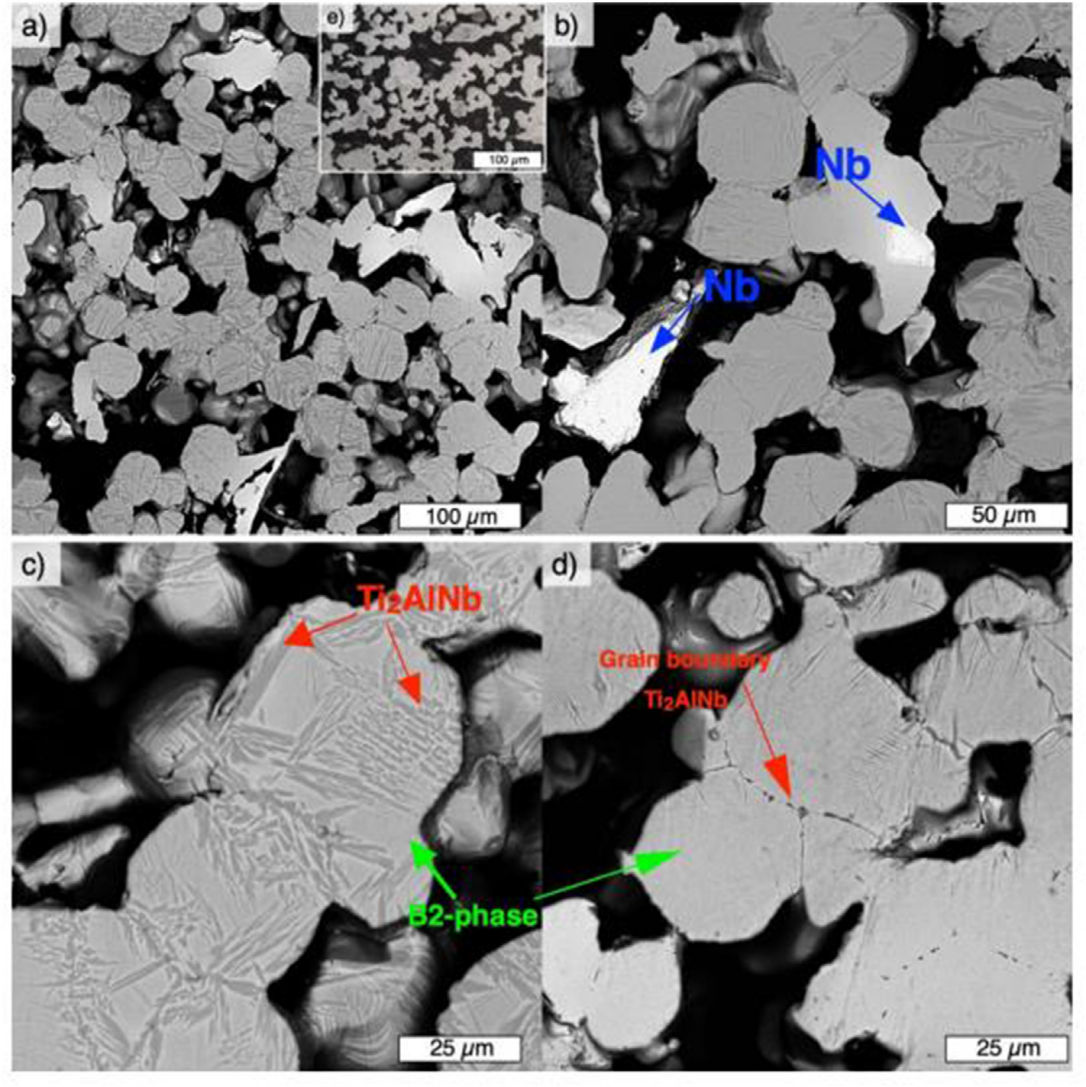
SEM micrographs of samples after annealing at 1400 °C for 2 h [94].

Polozov et al. [94] synthesized orthorhombic titanium alloy using 3D printing technique. Commercially pure titanium, aluminium, and vanadium were turbula mixed and printed using ExOne Innovent printer and ExOne solvent binder. The specimens were printed with a recount speed, binder saturation, and dry time of 15 s, 45% and 7 mm/s, respectively. Furthermore, the green parts were subjected to curing for 3 h at 180 °C, after which they were reactive sintered at 800, 1000 and 1110 °C in vacuum for 6 h however, the surface morphology of the samples was modified upon increase in sintering temperature from 1000 to 1100 °C, with smoother and reduced globular characteristics on the surface. The authors ascribed this modification to a change in the wettability property of aluminium at increased temperature. The XRD analysis (Figure 5) further revealed the existence of Ti_3_Al phase. The titanium atoms were reportedly dissolved on the surface of the molten aluminium after the specimens were heated above the melting temperature of aluminium. This resulted in the formation of enriched titanium solid solution, which later solidified as globules. Upon annealing of the sintered samples at 1400 °C, there was diffusion and dissolution of Ti, Al, and Nb, leading to the formation of solid solution and precipitates of Ti_2_AlNb phase. The SEM images of the after annealing shown in Figure 6 also reveals that the Ti_2_AlNb precipitates were formed within B2 grains and grain boundaries due to the annealing temperature adopted.

### 2.4 Selective laser melting (SLM)

**Figure 7:**
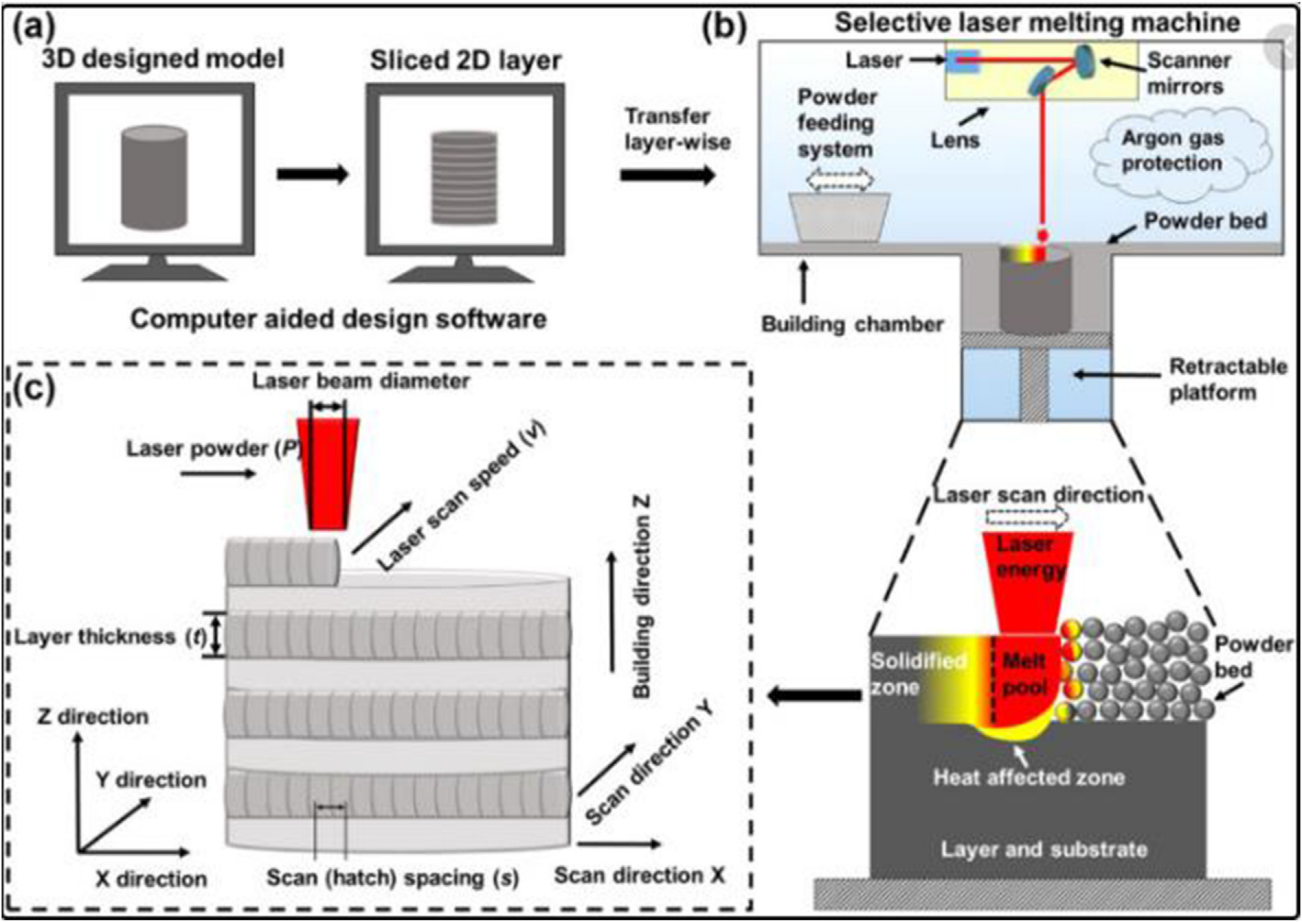
Schematic diagram showing (a) first, (b) second, and (c) third stage of the selective laser melting process [100].

Selective laser melting has been widely adopted for the fabrication of aerospace components using nickel-based superalloys, steels, and titanium-based alloy materials [95, 96]. This technique turns prototypes into functional hardware fabricated from the same material as production components. It also allows the design of organic geometrics and parts with challenging passages and internal features that could not be produced via casting and other conventional fabricating techniques since the components are built in layers. The fabrication process is initiated by designing a 3D model from CAD (computer-aided design) software. The optimized 3D model is mathematically divided into several 2D layers using specific AM software. The data generated from the 2D model is fed into the SLM computer, which assigns pre-defined scanning strategies and parameters. Prior to printing, it is essential to pressurize the building chamber with inert gas (nitrogen or argon) to prevent oxidation and contamination during the fabrication process. A schematic illustration of the selective laser melting process is shown in Figure 7 (a-c). However, the processing/scanning parameters should be carefully selected, as they play a crucial role in the solidification and melting process of the feedstock powders [97]. Improper melting/solidification can affect the microstructural properties of the fabricated components, thereby causing defects such as delamination, cracks, and pores. Moreover, energy density is an important parameter that is required to achieve high densification in fabricated components [98]. The laser energy density (E_d_) can be calculated using Eq. (1) [99].

(1) $$E_{d}=\;\frac{L_{p}}{L_{s}\;\times S_{s}}$$

where L_p_ is the laser power, L_s_ is the laser spot and S_s_ represents the scan speed.

A recent parametric study by Bhardwaj et al. [101] extensively analysed the microstructure and mechanical properties of titanium-molybdenum alloy, fabricated by laser AM technique. Spherical-shaped atomized molybdenum and titanium powder subjected to Hall flow test (according to ASTM B213) were used as the starting powders. The specimens were produced in an argon gas environment through a DED-LAM coaxial powder feeding nozzle. In this study, the authors adopted the laser power as the main parameter, and this was varied below 1300 and 1950 W. X-ray diffraction (XRD) analysis conducted confirmed the presence of a dominant β-phase planes (110), (220) and (211). The SEM images also showed the deposition of large columnar dendrites and several equiaxed grains. The microvoids generated in the specimens were attributed to gas entrapment, which occurred during the fabrication process. It should be noted that these porosities can be reduced/eliminated through post-processing treatment (hot isostatic pressing or heat treatment) and the adoption of different laser scans.

**Figure 8:**
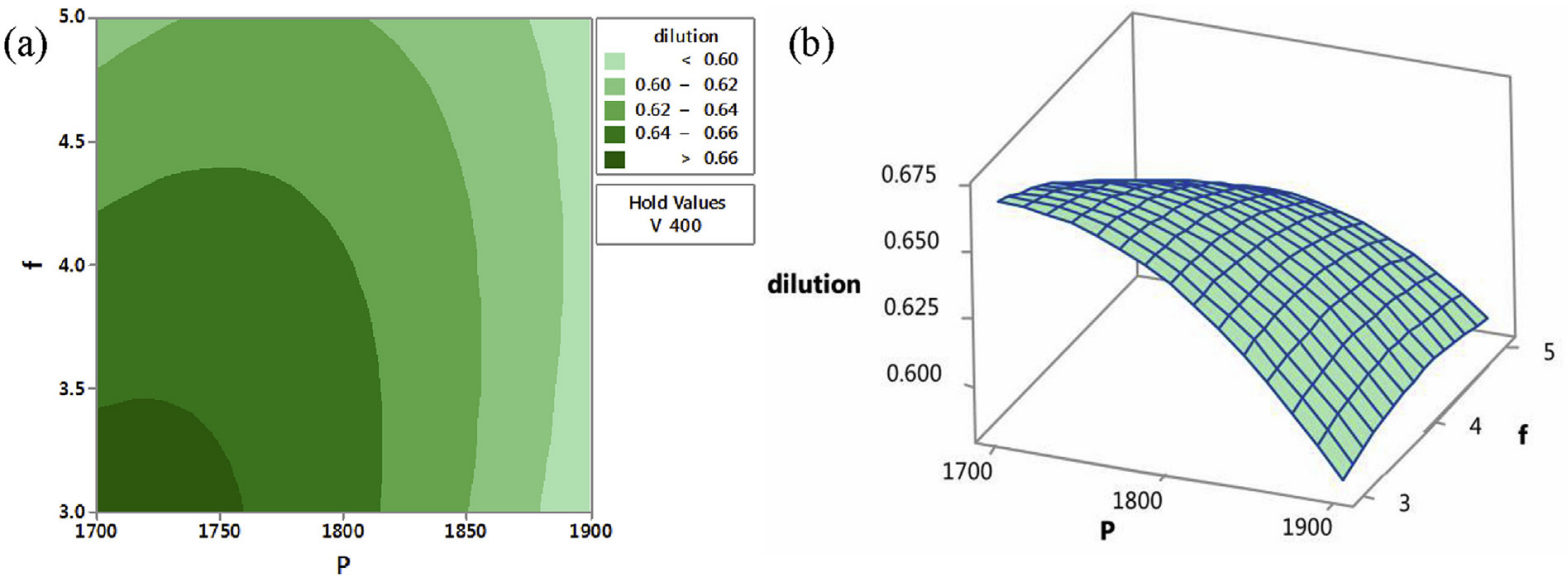
(a) Contour and (b) Surface plot of dilution (high laser power and feed rate at constant scan speed) [101].

**Figure 9:**
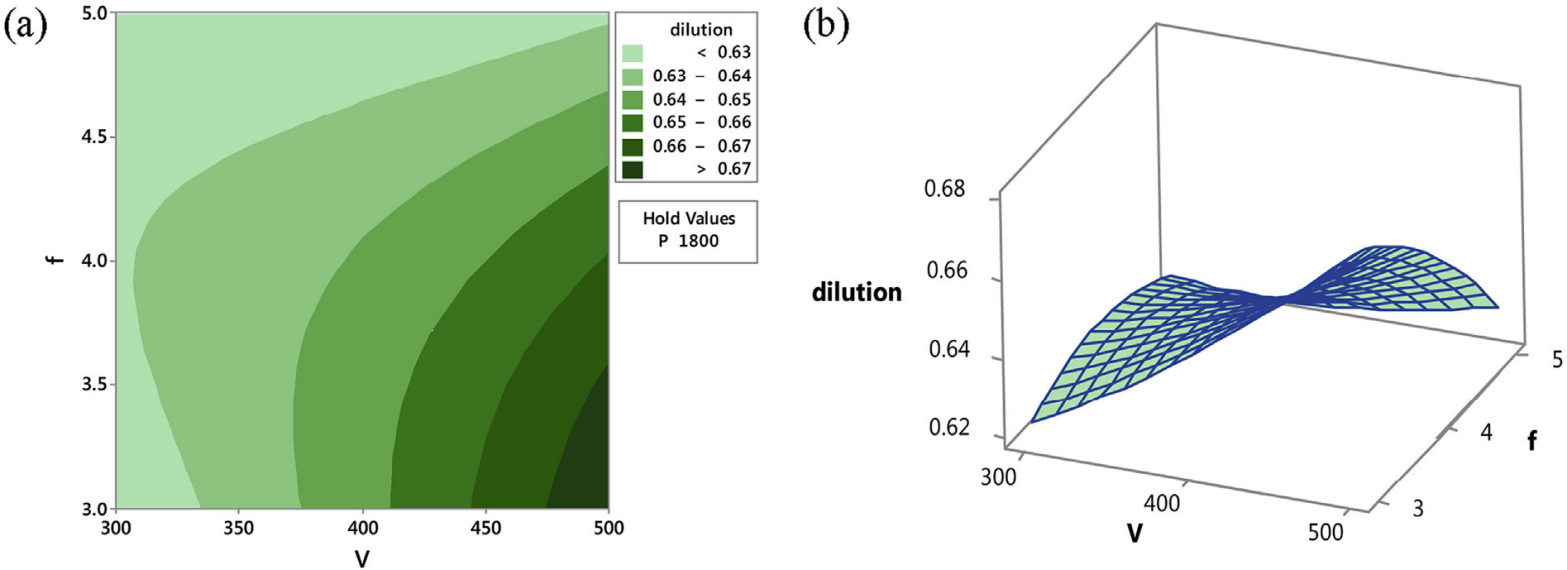
(a) Contour and (b) Surface plot of dilution (high scan speed and feed rate at constant laser power) [101].

Furthermore, track dilution was reported to be minimal at a low scan speed. A track dilution is dependent on track depth, and it is a function of track penetration and track bulge area. Figures 8 and 9 showed the contour and surface plots when minimum dilution was respectively observed at different parameters. This was noted at two different optimized parameters. The first was noted at a constant speed and high laser power, irrespective of the powder feed rate, while the second was achieved when the feed rate and scan speed were reduced with constant laser power.

The outstanding characteristic of all AM techniques is their ability to produce components of high geometrical complexity, which cannot be fabricated by any other conventional method. A detailed classification of different AM manufacturing techniques is presented in Table 2.

**Table 2:** Classifications of AM techniques.

Method	Various technology	Advantages	Limitations	Fabricated components	Ref
Directed Energy deposition	• Laser engineered net shaping • Electron beam direct melting • Rapid plasma deposition • Direct light fabrication • 3D laser cladding	• Ability to control the grain structures • Production of relatively large parts with minimal tooling • repair of high-quality functional parts	• Surface finishing is dependent on the material used • Post-processing finishing is needed to achieve the desired effect	• Polymers • Metals • Composites	[102, 103]
Binder jetting	• Ink jetting • 3D printing • Color jet printing	• Ease of building parts in multiple materials • Relatively low cost of fabrication • High speed • Allows the combination of powder materials and additives binders	• Poor accuracy and surface finish • Infiltration steps are required to achieve good mechanical properties	• Metals • Ceramics	[104, 105]
Powder bed fusion	• Selective laser melting • Direct metal laser sintering • Selective laser sintering	• Improved production development time • Efficient for reduced volume production and rapid prototypingEfficient recycling of un-melted powder	• Requires post-processing • Longer print time and relatively low speed of production	• Ceramics • Glass • Plastic • Metals	[106, 107]
Vat polymerization	• Digital light processing technology • Stereolithography	• High level of accuracy and excellent surface finish • It is relatively fast • Has large build areas	• It can only be used for photo-resin materials • After print, components are affected by ultraviolet rays • Longer processing time.	• Polymer • Ceramics	[108, 109, 110]
Material jetting	• Thermojet • Polyject	• Parts are useful for the design of casting pattern • Excellent accuracy and surface finishes	• Fragile parts owing to wax-like materials • Relatively slow build process	• Photopolymer • Ceramics	[110, 111]
Sheet lamination	• Ultrasonic AM • Laminated object manufacturing	• Ease of material handling • Support structures are not required • Relatively cost-efficient	• More waste is generated in comparison to other AM techniques • It has limited material options • Not suitable for the fabrication of hollow parts	• Metals • Ceramics	[112, 113]

## 3 Microstructural properties of 3D printed Ti-based alloys

**Figure 10:**
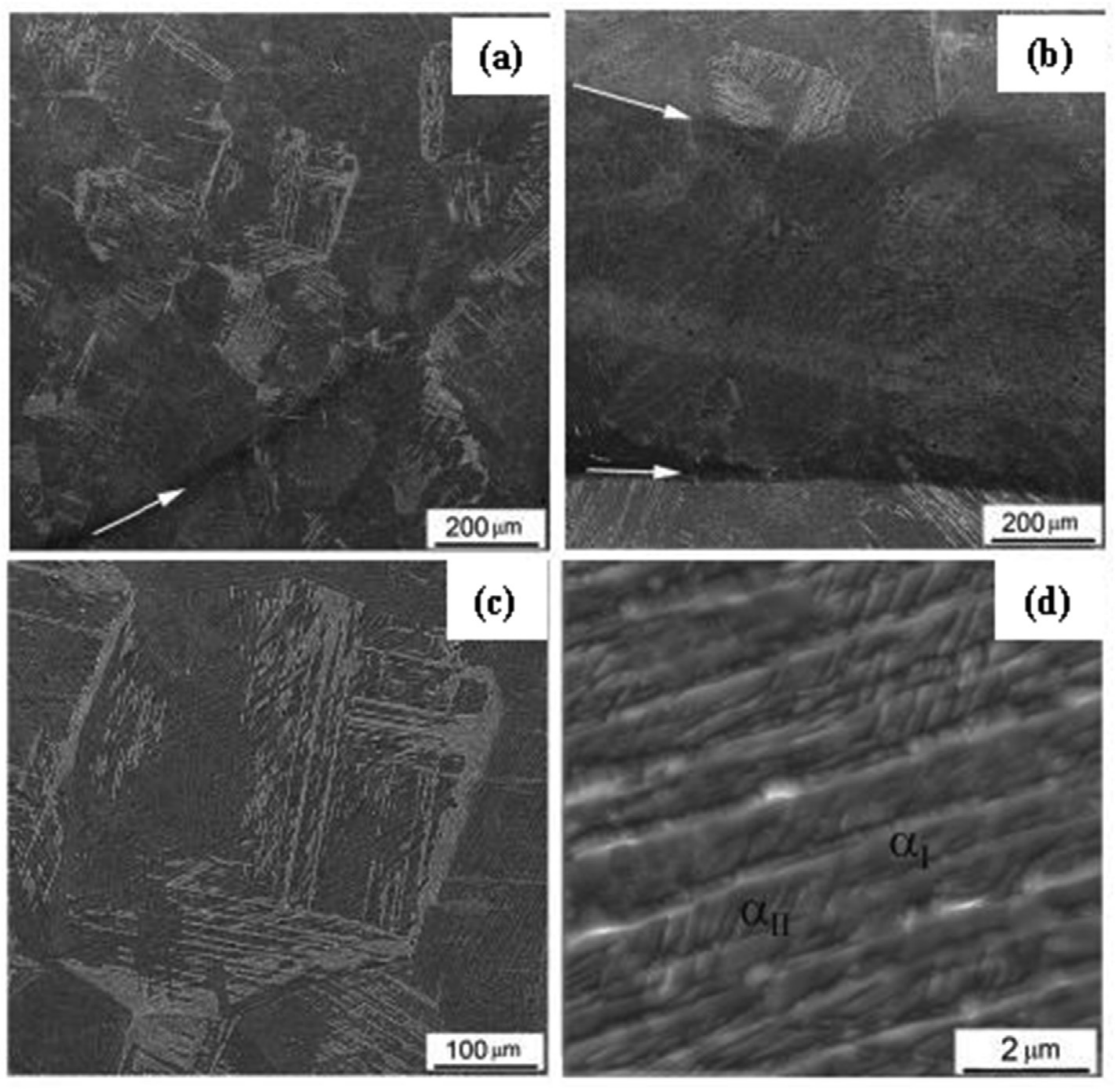
SEM micrographs of Ti–6Al–4V material in directions (a) X (b) Y (c) Z and (d) secondary α-phase crystal between coarse α plates [114].

**Figure 11:**
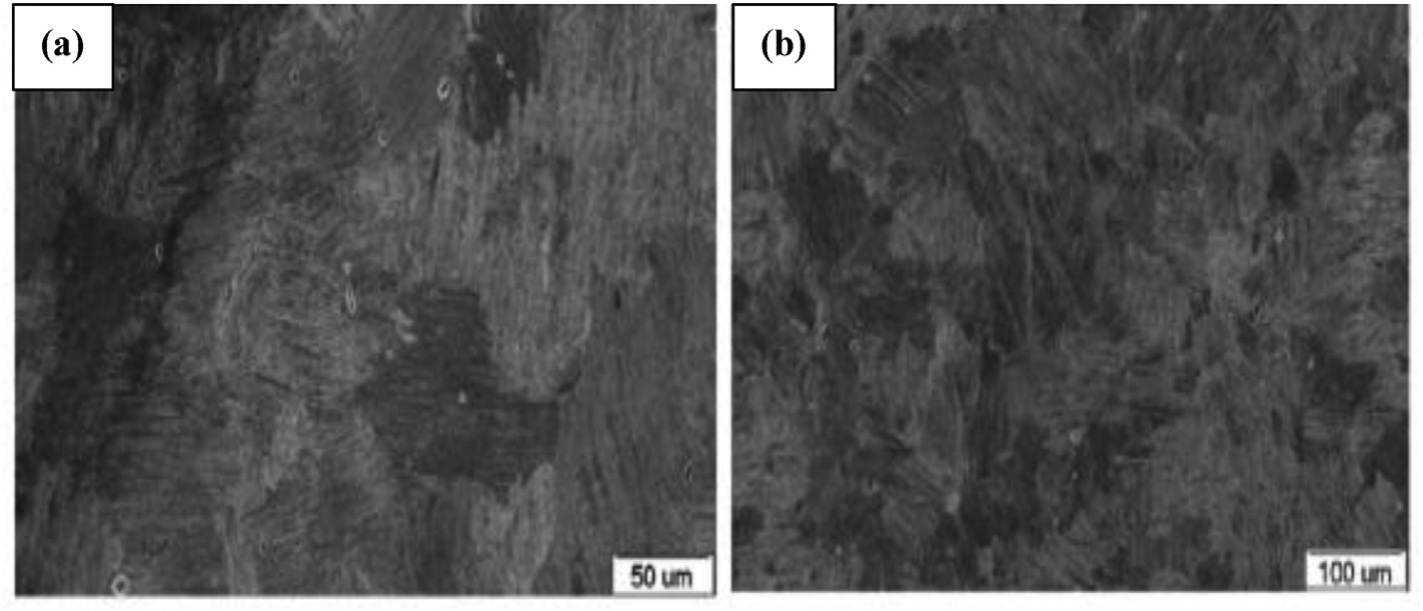
Microstructure of the as-deposited (a) Ti–47Al–2.5V–1Cr and (b) Ti–40Al–2Cr alloys [115].

**Figure 12:**
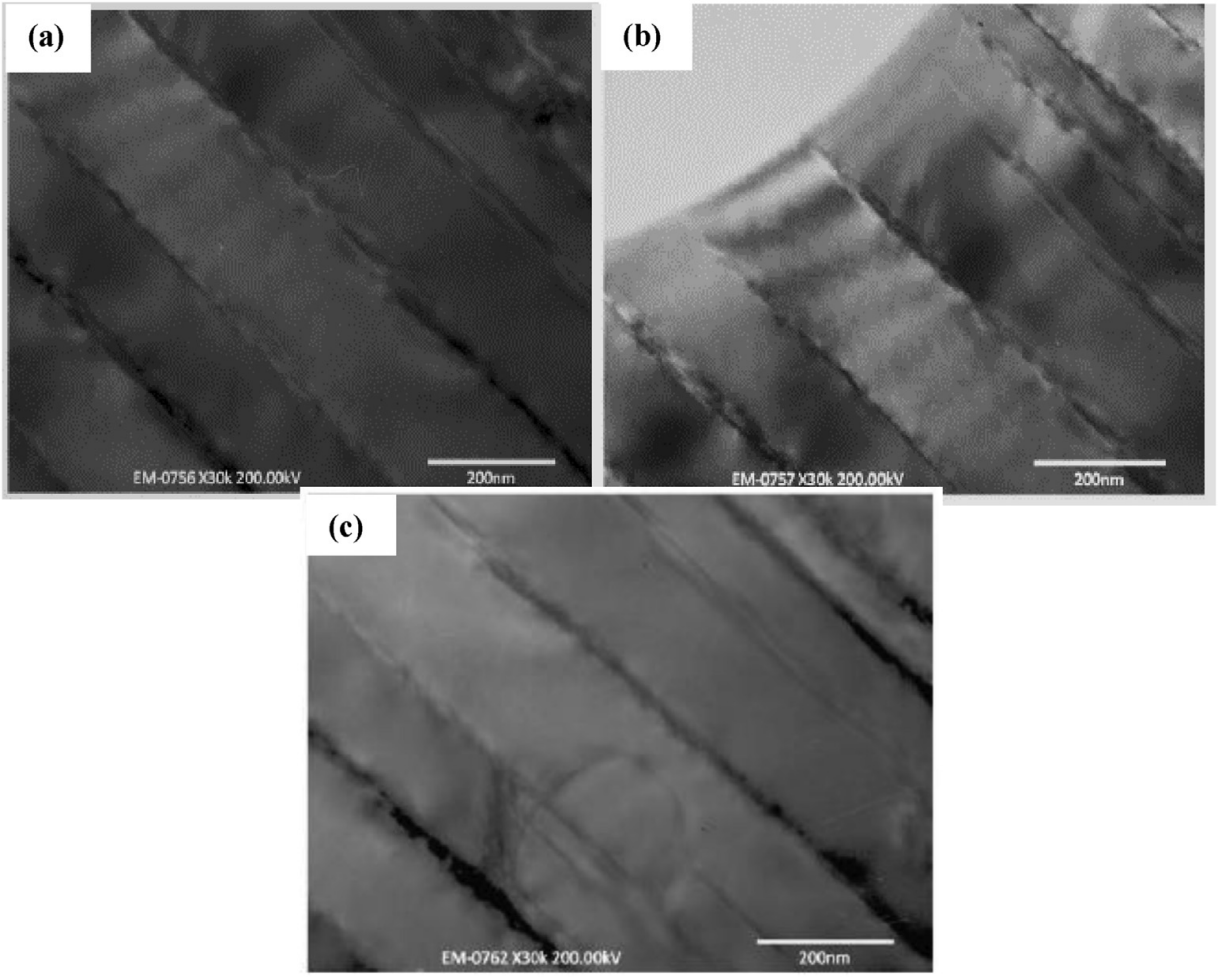
TEM images of the heat-treated TiAlCr alloy at (a) 1100°C/30 min (b) 1100°C/30 min (c) 1125°C/30 min [115].

AM is known for its ability to produce site-specific microstructures. It can fabricate components through a continuous layer addition, with a thickness less than ten microns. Owing to this, the microstructural feature of a 3D printed engineering material plays a crucial role in determining its physicochemical and chemical properties. It is also noteworthy that factors that include thermal gradient and rate of solidification help control the microstructural properties of fabricated materials. These factors, however, determine the arrangement of grains, defects, and crystallographic structure of the fabricated material. In a recent study by Kovalchuk et al. [114], the SEM morphology of a 3D printed Ti–6Al–4V article after heat treatment was investigated, and shown in Figure 10. The β-grain structure is seen to cross the boundaries between the vertical (Figure 10a), and the horizontal (Figure 10b) layers. The α-colonies gave an intergranular shaped structure (Figure 10c). The crystals dispersed in the secondary α-phase at high magnification is presented in Figure 10d. The Qu et al. [115] discussed the microstructural features of Ti–47Al–2.5V–1Cr and Ti–40Al–2Cr (at.%) alloys fabricated by LMD process. Results from this investigation showed a fully lamellar microstructure of different grain size consisting of α_2_-Ti_3_Al and γ-TiAl phases. The fine lamellar structures seen in the as-deposited TiAl specimens was attributed to high solidification rate during the manufacturing process. However, the lamellar microstructure which consists of γ-TiAl and α_2_-Ti_3_Al as seen in Figure 11a and b reportedly resulted from the solid-state phase transformation. This study showed no clear indication of surface morphologies to ascertain if the cracks seen on the microstructure resulted from the high solidification rate used during fabrication. Figure 12 shows the TEM images of the heat-treated alloys. From Figure 12a, there is a clear indication that the initial crack was generated as a single grain, which later propagated along with the lamellar interface and grain boundary. However, Figure 12b and c revealed that the lamellae spacing is on the same level, resulting from the short duration used during heat treatment.

Wang et al. [116] also reported on the mechanical and microstructural homogeneity of Ti–35Nb alloy, fabricated via selective laser melted method. The SEM images from this study revealed the presence of dendritic niobium particles dispersed within the Ti–Nb matrix. This however indicates that the molten pool underwent a rapid cooling, and the laser powder had a reduced interaction time, which resulted in incomplete diffusion and dissolution of the niobium particles.

**Figure 13:**
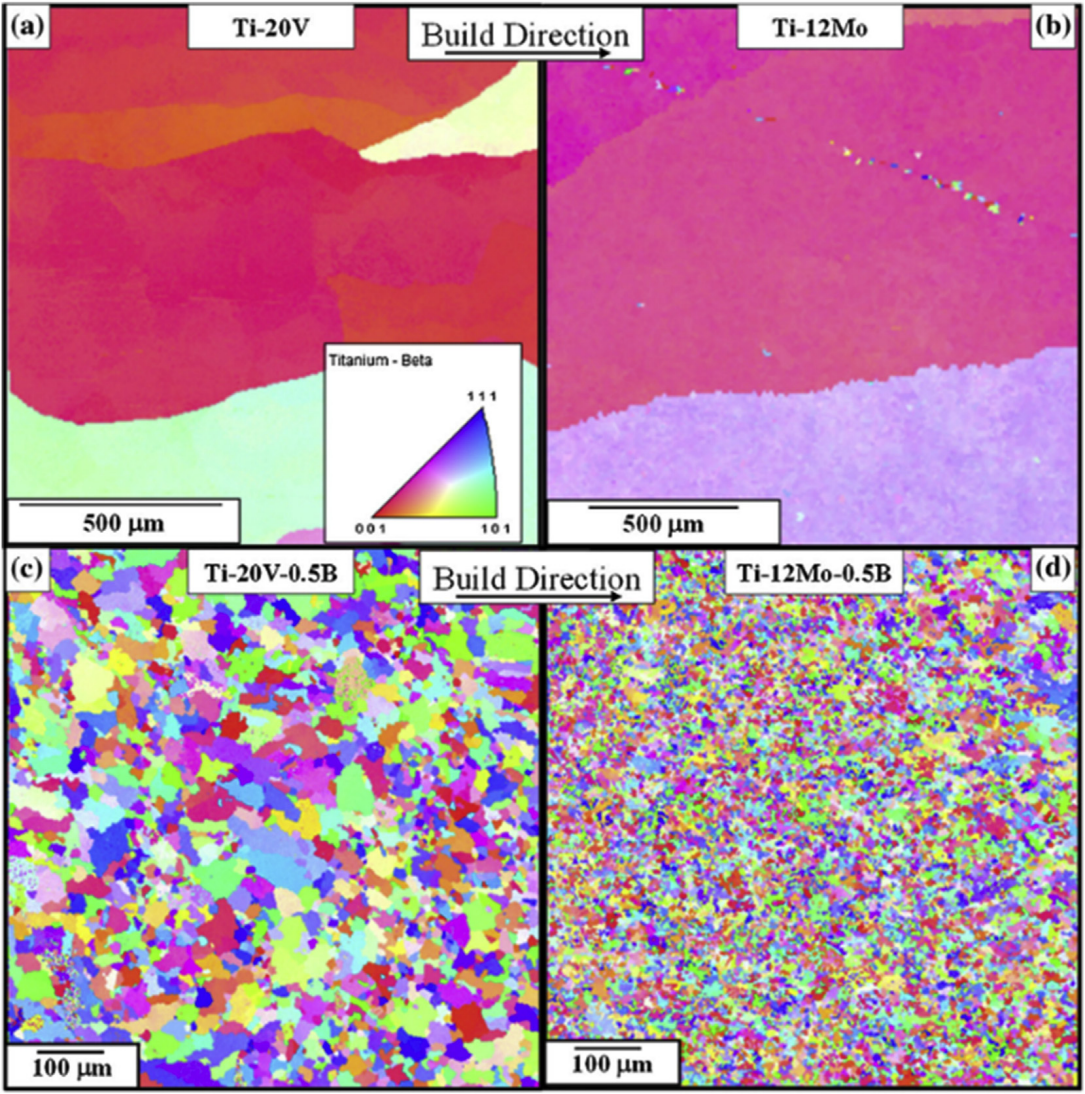
EBSD-IPF maps for (a) Ti–V (b) Ti–Mo (c) Ti–V–B (d) Ti–Mo–B alloys [117].

The effect of boron addition on the texture and grain size of additively manufactured β-Ti alloys was examined by Mantri et al. [117]. This study was reportedly motivated by the need for a detailed microstructural and textural investigation in additively manufactured components, which is a function of spatial location and a model based on the thermomechanical history as well as development of approach to microstructural consistency, and good understanding of the resulting texture in additively manufactured TiAl alloys. Different titanium-based powders were deposited through laser engineered net shaping (LENS) technique, from a blend of high purity feedstock powders of titanium (Ti), vanadium (V), molybdenum (M), and boron (B). The printed specimens were sectioned in the middle, and their surfaces were prepared for microstructural examination using standard metallography procedures. Electron backscattered diffraction (EBSD) analysis was performed on the specimens to get detailed information on their texture and grain sizes. The EBSD Inverse Pole Figures (IPF) maps are presented in Figure 13 (a-d). The EBSD scans were reportedly carried out across different regions within the specimens. A major observation from this study revealed that the grains exhibited columnar growth, which do not exceed 2 mm along the long axis (Figure 13a and b). However, the addition of boron (Figure 13c and d) to the Ti-based powders mapped out the grains, which illustrates grain modification.

**Figure 14:**
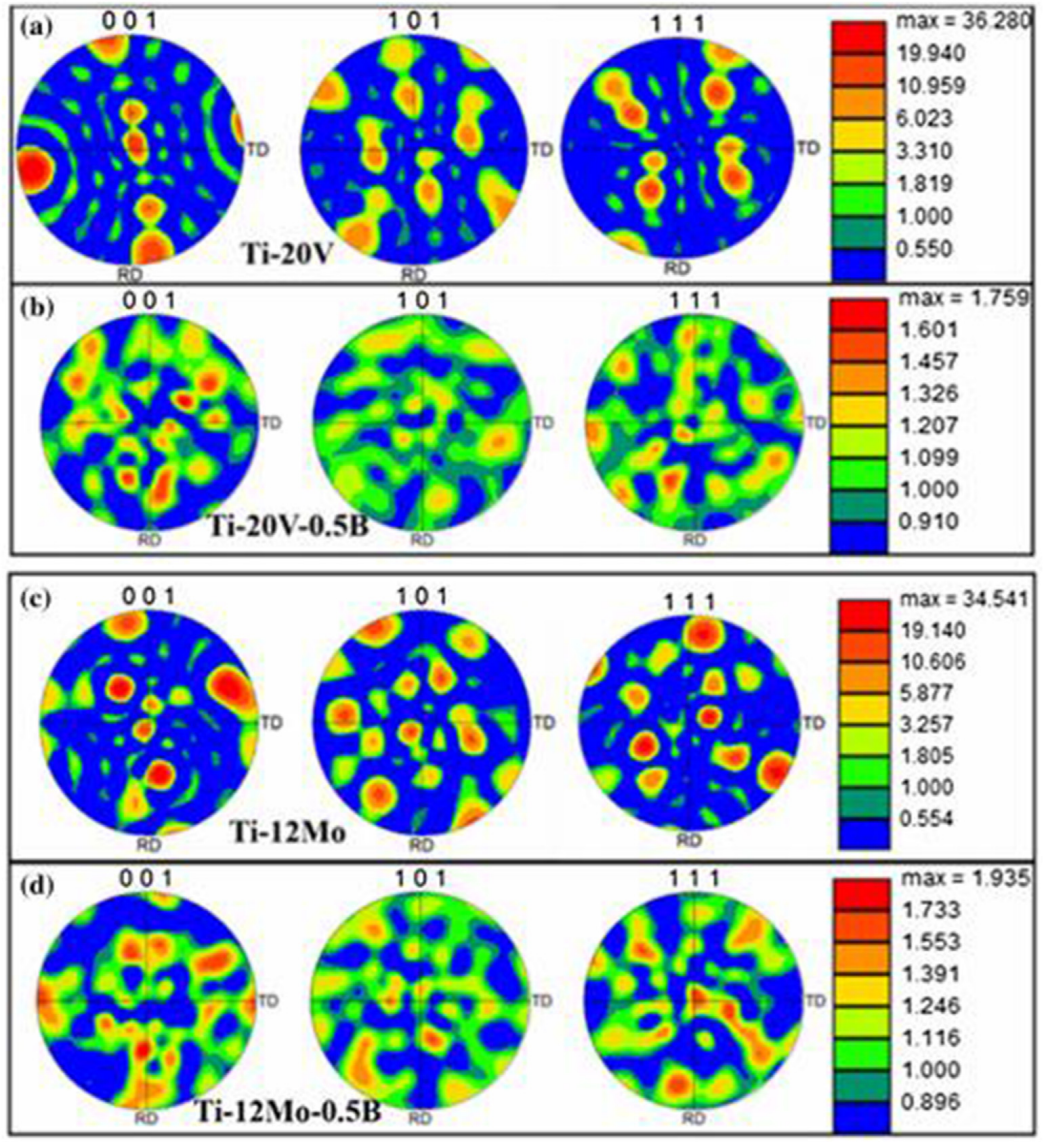
Texture maps of (a) Ti-20 wt% V (b) Ti-20 wt% V-0.5 wt% (c) Ti-12 wt% Mo and (d) Ti-12 wt% Mo-0.5 wt% B [117].

The texture maps of the specimens are shown in Figure 14. The authors reported only on the texture maps from the β grains since the texture is developed during the powder deposition stage. The Ti–V and Ti–Mo specimens exhibited the maximum texture intensity (MTI) values of 37 and 32, respectively. The high MTI values indicate the growth of a strong (001) β columnar grain along the cylindrical deposit axis (Figure 14a and c). Furthermore, incorporating boron into the Ti-Vand Ti–Mo powders resulted in decreased MTI values of 1.75 and 1.9, respectively (Figure 14b and d), thereby developing random textures.

Also, Löber et al. [118] investigated the influence of different processing parameters on the fabrication of beta solidifying titanium aluminide (Ti–Al28.9–Nb9.68–Mo2.26–B0.024) alloy. In this study, the authors adopted laser beam power ranging from 50 to 250W and scanning speeds within the range of 50–2100 mm/s during fabrication. The results obtained showed the effect of the scanning speed and the laser power on the morphology of the melt tracks. Furthermore, at an intermediate laser power (100 W) and very high scanning speeds (350–2100 mm/s), a balling phenomenon was observed.

Balling, which often results from discontinuous and disconnected scan tracks, is a significant defect associated with the direct metal laser sintering technique [119, 120]. It further inhibits even deposition of new powder layer, thereby leading to the formation of pores and delamination initiation because of poor bond strength between layers. Another reason for balling effect has been ascribed to the presence of oxide contamination on the melt and substrate surface.

**Figure 15:**
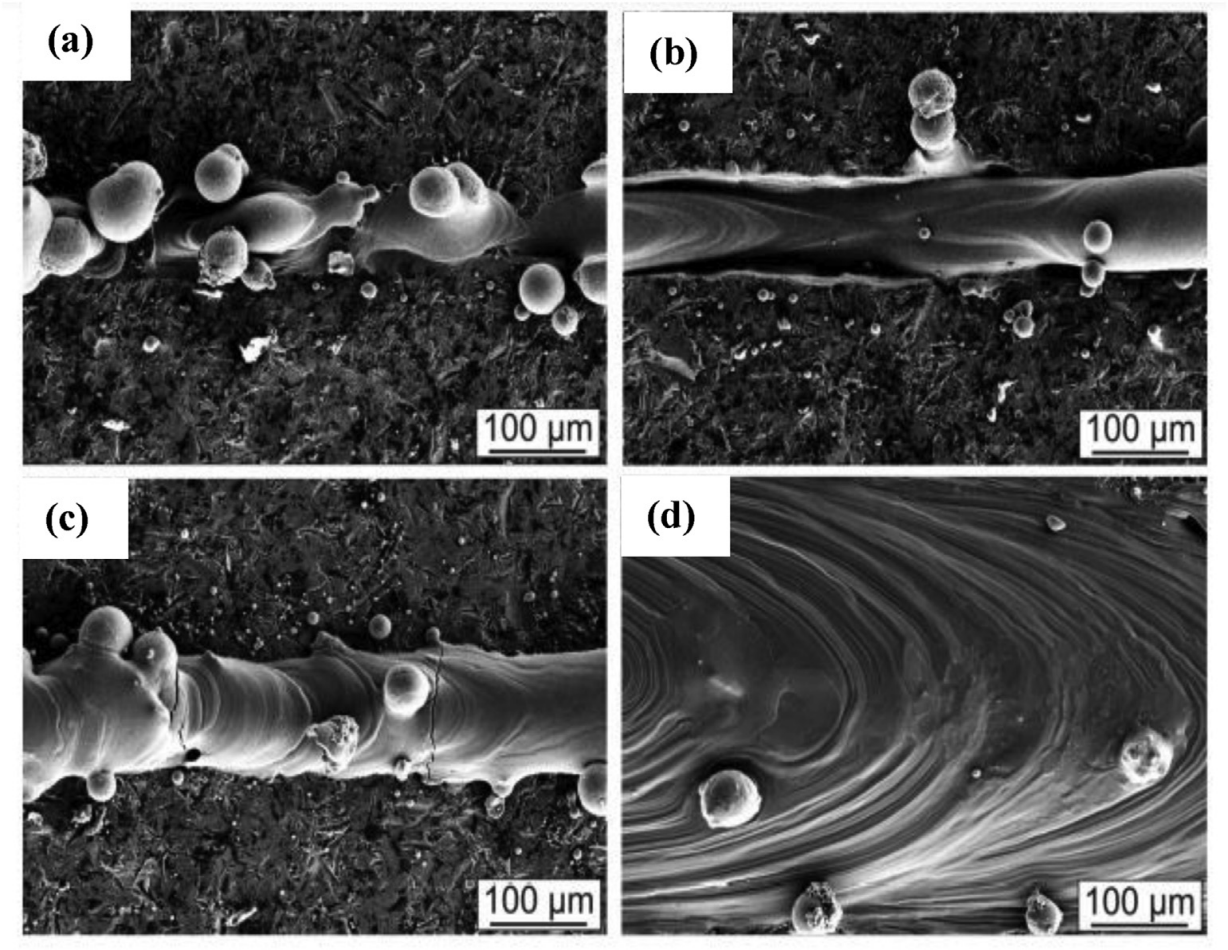
Microstructural examination of specimens showing (a) balling, (b) unstable melt track, (c) melt track with cracks, and (d) stable melt track [121].

The study further confirmed that maintaining higher scanning speeds and power altered the microstructure from balling to an unstable melt track, as seen in Figure 15 (a-c). The last morphology was observed to a stable and smooth melt track when slow scanning speeds (50–100 mm/s) and intermediate to high laser powers (100–250W) processing parameters were adopted (Figure 15d).

In view of all that has been mentioned so far, it is essential to note that the LMP addictive manufacturing technique may become a preferred method for the fabrication of TiAl alloys with finer grain sizes, resulting in enhanced mechanical properties.

## 4 Mechanical properties of 3D printed Ti-based alloys

Additively manufactured titanium alloys have been reportedly subjected to various types of mechanical testing to better understand their mechanical behaviour under severe service conditions. Researchers have extensively used tests that include but not limited to tensile, hardness and tribology to ascertain the mechanical properties of the fabricated alloys.

The effect of boron addition on the resistance of additively manufactured titanium alloy to wear deformation was studied by Mantri et al. [14]. This research was reportedly motivated by the need to use titanium-based alloys in the biomedical engineering industry due to their improved corrosion and fatigue resistance and their reduced elastic modulus [15, 16]. The insolubility of boron in titanium when in solid state has been reported, and this often prevent embrittlement within titanium matrix. Murr et al. [32] compared the mechanical and microstructural properties of Ti–6Al–4V fabricated by the electron beam melting (EBM) process to wrought and cast fabricated Ti–6Al–4V samples. Findings from this research show that the investigated AM techniques are more efficient for the production of biomedical implant components, with superior mechanical properties, compared to the cast and wrought fabricated Ti–6Al–4V components.

In a similar investigation by Rafi et al. [122], two different 3D printing techniques were compared (selective laser melting and electron beam melting) based on the microstructural and mechanical properties exhibited by the fabricated Ti6Al4V parts. Their results showed that the Ti64 sample produced via SLM technique recorded a higher tensile strength compared to the EBM-produced samples. However, the EBM-produced samples were reported to record an enhanced ductility as presented in Table 3. The authors attributed the higher tensile strength shown by the SLM samples to its martensitic microstructure, while the higher ductility in EBM-produced samples was ascribed to the presence of a lamellar phase. Moreover, a fatique limit of 500 MPa was recorded by the samples fabricated by SLM technique. In contrast, the EBM-produced samples had a fatigue limit of 340 MPa, and this behaviour was ascribed to the presence of lamellae structures revealed in their micrographs.

**Table 3:** Tensile results for SLM-produced and EBM-produced Ti64 alloy samples [122].

	Stress at yield (MPa)	Ultimate tensile stress (MPa)	Strain at break (%)
EBM (vertically built and machined)	869	928	9.9
SLM (vertically built and machined)	1143	1219	4.89
EBM (horizontally built and machined)	899	978	9.5
SLM (horizontally built and machined)	1195	1269	5
Percentage increase	33	30	47

**Figure 16:**
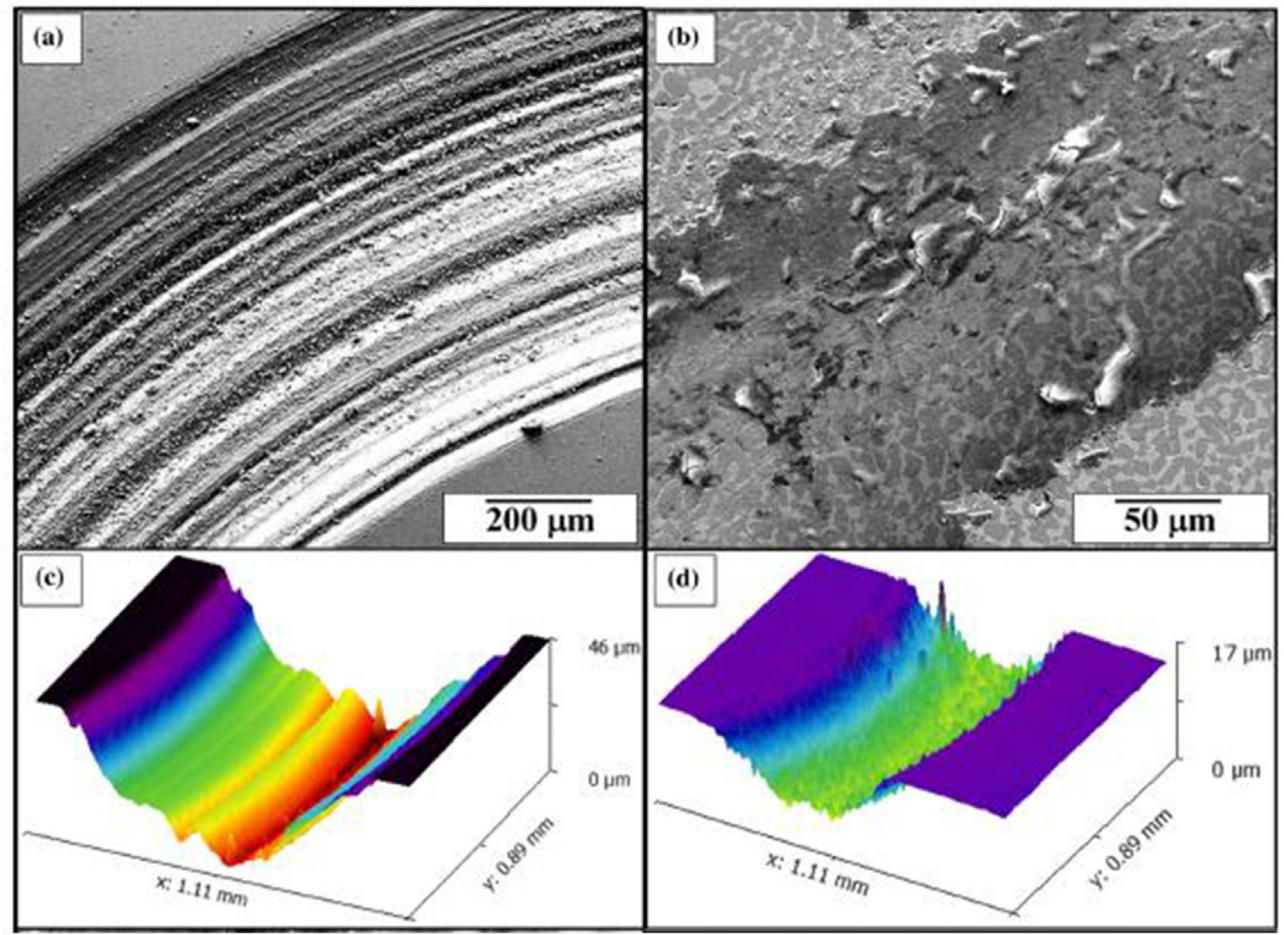
(a–b) SEM and (c–d) SWLI images for wear tracks of TNZ and TNZ-B respectively [123].

In another study by Mantri et al. [123], the influence of boron reinforcement on the hardness and wear properties of 3D printed titanium-based alloy was investigated. Two specimens with the label TNZ (with composition Ti–13Nb–13Zr) and TNZ-B were reportedly examined. The hardness property was evaluated under a load of 2000 mN for 10 s, while the wear behaviour was carried out using a silicon nitride counterface and a static load of 1N, which corresponds to an initial mean Hertisan contact stress of 500 MPa. Findings from this investigation show that the TNZ specimen recorded an overall hardness value of 325 HV, which is a bit higher than the values reported in the literature [124, 125]. This behaviour was reportedly ascribed to fine grains and residual stress, which resulted from the laser-engineered net shaping (LENS) printing technique adopted for the fabrication of the specimens. Upon incorporating boron into TNZ, the hardness value of TNZ-B increased to an average value of 450 HV. The authors attributed the increase in hardness value to the formation of in-situ TiB precipitates. Moreover, the TNZ-B showed a reduced wear rate compared to the TNZ alloy due to the increased hardness, which was previously reported to result from the formation of TiB and the change in volume fraction and morphology of α precipitates. The images seen in Figure 16a and b reveal the SEM micrographs of the wear tracks generated on both specimens, while their 3D Scanning White-Light Interferometer (SWLI) images are presented in Figure 16c and d. Figure 16c depicts an increase in the wear track width (−850 μm) and depth (−46 μm) generated by the TNZ and TNZ-B alloys, respectively.

Conversely, Figure 15d showed a wear track width of -150 μm and a depth of -17 μm for the TNZ-B. In conclusion, the TNZ alloy exhibited microploughing and extensive plastic deformation, which depicts abrasive wear. However, fewer of these wear mechanisms were evident in TNZ-B alloy.

**Figure 17:**
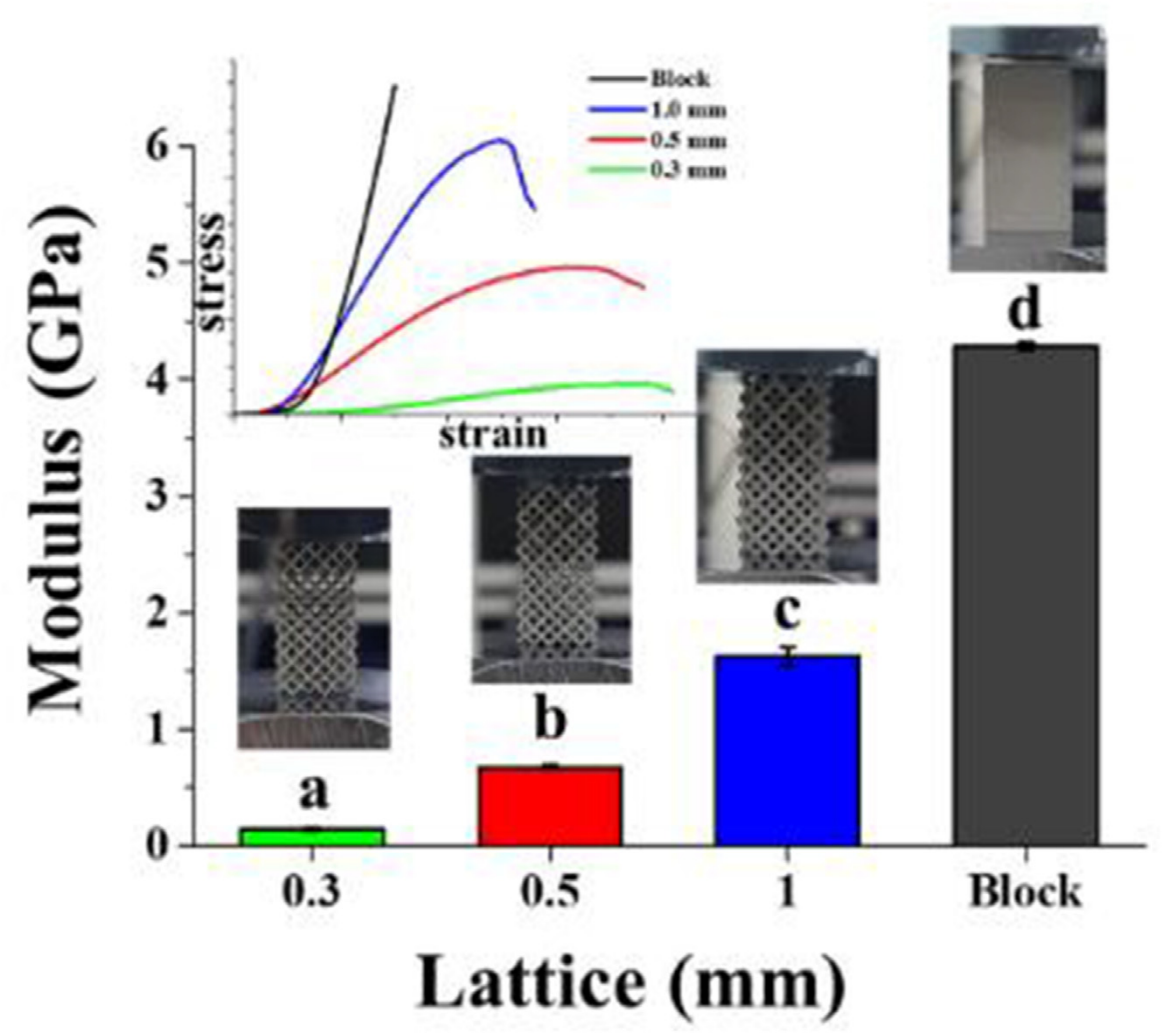
Elastic modulus modulation in fabricated titanium alloy [126].

Kim et al. [126] carried out extensive research on the biomedical properties of 3D printed titanium-based alloy for dental application. The specimens were produced using a direct laser metal sintering technique in an argon atmosphere. The bar specimen analysed for mechanical properties were prepared at fabricated at different span width of 1, 0.5, and 0.3mm, and laser spacing of 90, 70, 50and 30 μm. In addition, the authors carried out a comparison between the elastic modulus of the specimens and a dense block. It was reported that the specimen with 0.3–0.1 μm lattice yielded a reduced modulus with increased flexibility. Figure 17 represents the analysis, with the different alphabets denoting a major difference between the specimens (n = 3, P < 5). This study shows that the mechanical properties of the fabricated specimens are affected by the laser spacing adopted for printing.

**Figure 18:**
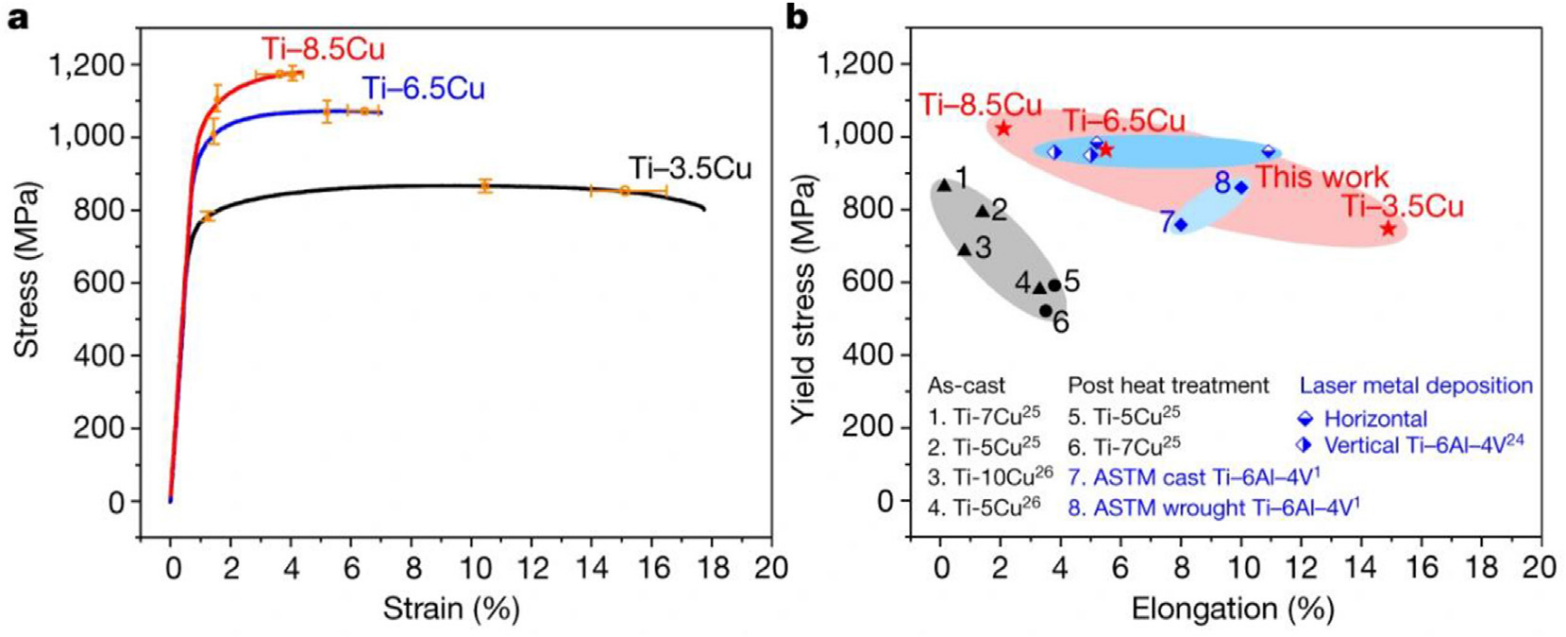
(a) Stress-strain curves of fabricated samples (b) Plot of yield strength vs tensile elongation for Ti–Cu alloy produced via different fabricating techniques [127].

Zhang et al. [127] reported their findings on AM high-strength titanium -copper (Ti–Cu) alloys with ultrafine grains. The authors maintain that the dimension of laser melted regions and increased thermal gradient in titanium alloys suppresses the supercooling rate, thereby making the formation of fine grain size challenging. Ti–Cu alloy samples (Ti-3.5Cu, Ti-6.5Cu, Ti-8.5Cu) were fabricated using the laser melted deposition technique. Ti–6Al–4V specimen was also fabricated using the same parameters adopted for producing the Ti–Cu alloy for comparison. For the tensile test, cuboid samples were machined to tensile dimension, and the loading direction was reportedly perpendicular to the building direction used during laser melting deposition. The authors found that the Ti-6.5Cu exhibited an increased strength with reduced ductility (Figure 18a). Comparing the Ti-6.5Cu and Ti-8.5Cu, the latter recorded an improved strength, and this was ascribed to an increased volume fraction of eutectoid lamellae. The reduced ductility observed in the alloy with a high-volume fraction of Cu was attributed to the formation of hyper-eutectoid Ti_2_Cu compound. Furthermore, the Ti–Cu alloy fabricated via AM technique was compared with post heat treatment and casting specimens. The 3D printed Ti–Cu alloy is seen in Figure 18b to display the combination of superior ductility and yield strength.

## 5 Corrosion of titanium and titanium alloys

Several reports have shown that titanium is a reactive metal, which forms passivating oxide layers like stainless steel, in addition to aluminium and nickel-based alloys when exposed to aerated and aqueous environments [128, 129, 130, 131, 132]. In contrast to other metals, titanium and its alloys can undergo corrosion slowly or fast, depending on the environmental condition [133, 134]. Despite their overall enhanced corrosion, they still suffer corrosion attacks, which could be any combination of uniform corrosion, hydrogen embrittlement, pitting corrosion, erosion corrosion, and sometimes stress corrosion cracking [135, 136, 137]. Therefore, titanium supports a general rule which classifies corrosion as more of a system property than a material property (elastic modulus, electrical conductivity, and other physical properties).

Titanium becomes stable and energetic when it within an oxidation state of IV (TI^4+^), and will only exhibit oxidation states of II and III when an unstable and intermediate product is formed during the corrosion process. Eqs. (2) and (3) show the overall reaction of a metal (M) and titanium (Ti) in an aqueous environment (water), respectively [138].

(2) $$M+nH_{2}O\;↔M{\left(OH\right)}_{n}+\;\frac{n}{2}\;H_{2}$$

(3) $$Ti\;4H_{2}O\;↔Ti{\left(OH\right)}_{4}+2H_{2}O$$

**Figure 19:**
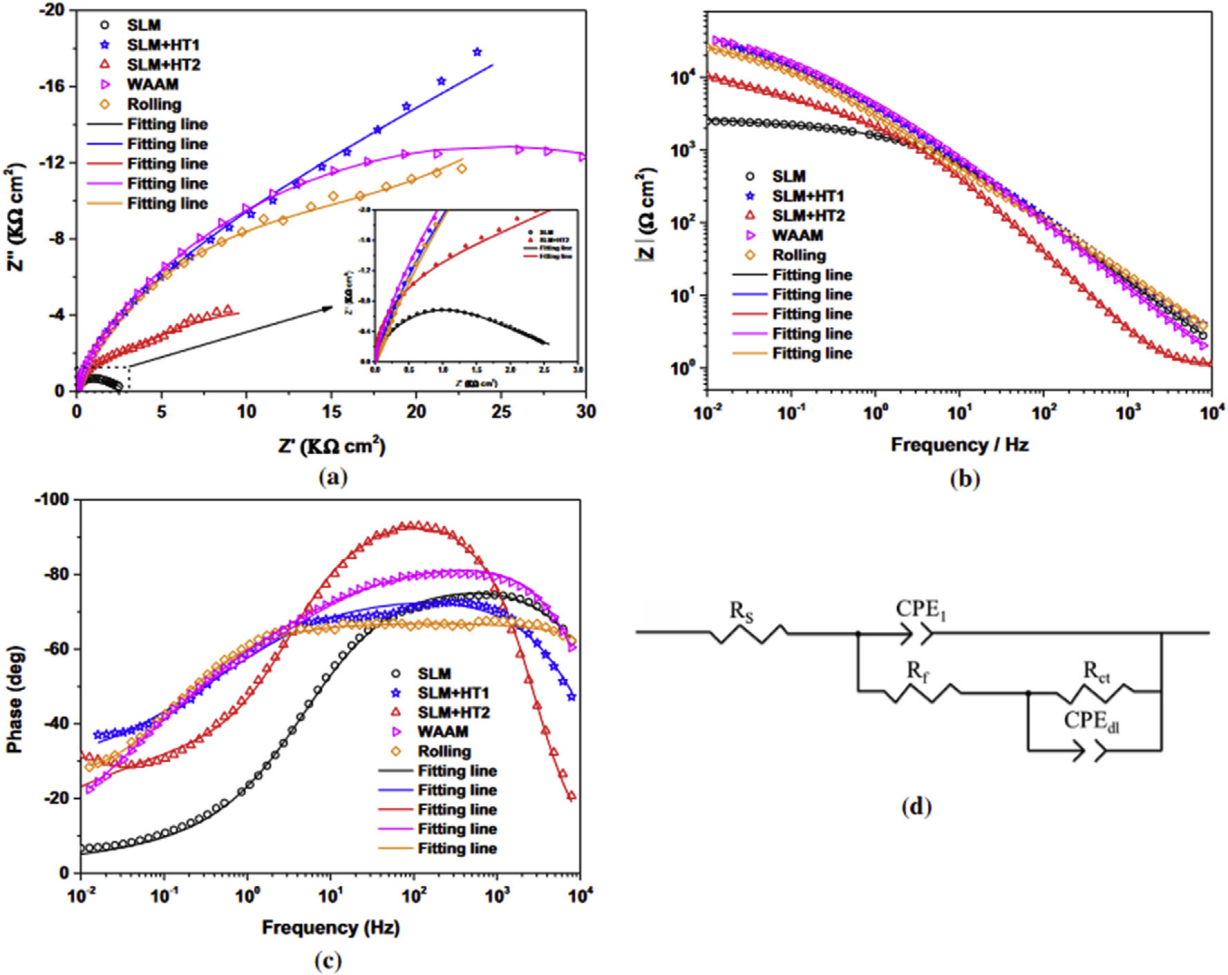
(a) Nyquist (b, c) Bode plots, and (d) equivalent circuit of Ti6Al4V specimens in sodium chloride electrolyte [144].

Recent studies conducted to evaluate the corrosion properties of additively manufactured titanium alloys showed that they exhibit a decreased corrosion resistance compared to titanium alloys fabricated using other conventional manufacturing techniques [139, 140]. This deterioration was ascribed to a decrease in the quantity of stable β-phase and increased metastable α-phase owing to rapid solidification of AM process. However, post-fabrication processing such as heat treatment and hot isostatic pressure has been employed for solving this challenge [141, 142, 143]. Yang et al. [144] examined in sodium chloride the corrosion resistance of different Ti6Al4V specimens from wire and arc manufacturing, SLM, heat-treated SLM, and traditional rolling techniques. From the potentiodynamic polarization test (PP), the heat-treated SLM specimen showed the least current density. Further analysis conducted using electro impedance spectroscopy (EIS) which also confirms the PP result, as the heat treated SLM specimen recorded the highest charge transfer (R_CT_), while the SLM specimen showed the least R_CT._ Result from this study also confirms the effectiveness of post-fabrication processing on specimens fabricated using selective laser melting technique. The Nyquist and Bode plots from the EIS test conducted is shown in Figure 19 (a-c).

**Figure 20:**
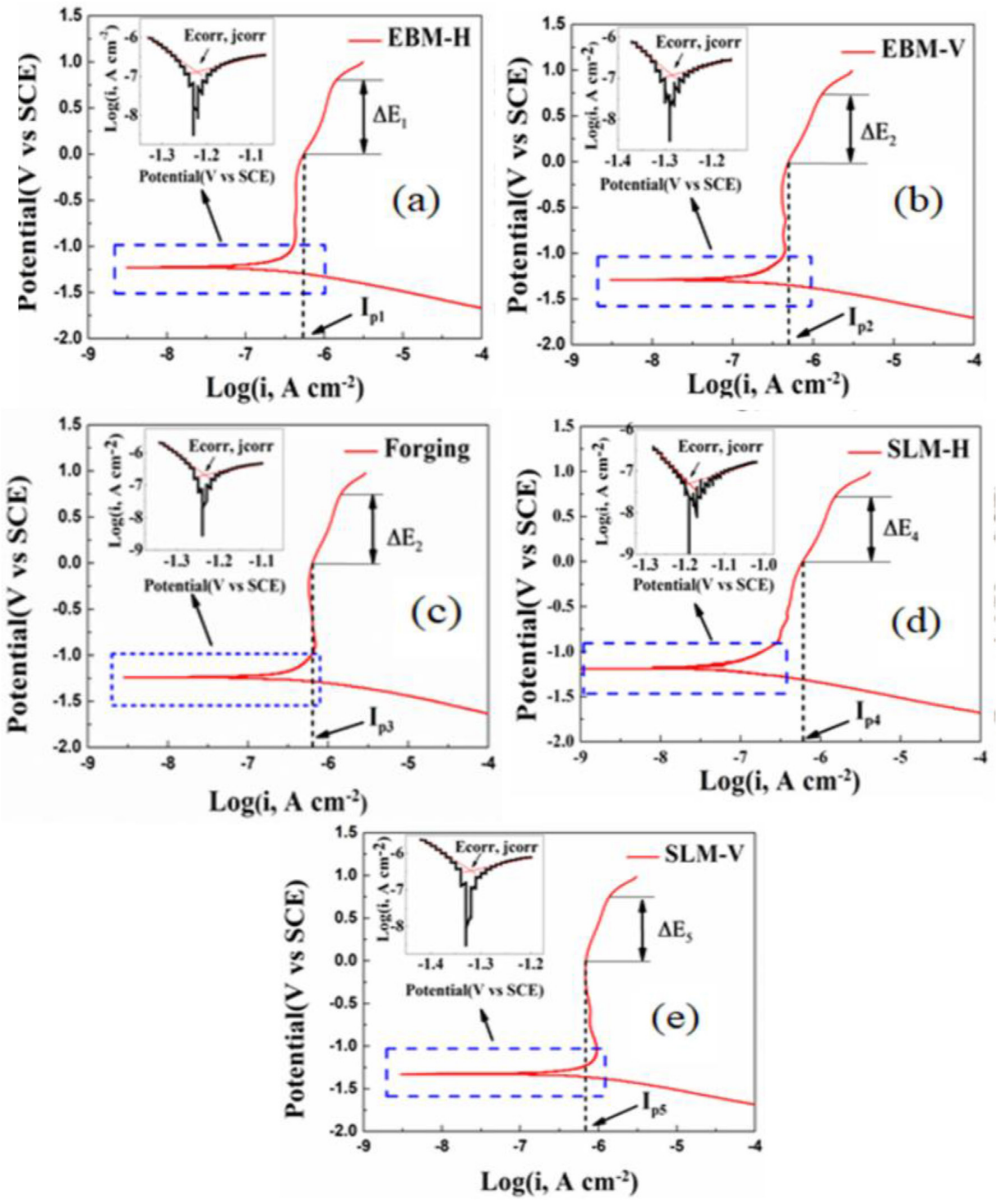
Potentiodynamic polarization and corresponding Tafel curves for (a) EBM-H, (b) EBM-V, (c) forging, (d) SLM-H, and (e) SLM-V alloys immersed in RDF in deicing electrolyte [146].

The corrosion properties of Ti–6Al–4V alloy fabricated using powder bed fusion method was investigated by Chen et al. [145]. A point defect model used in this study revealed that the Hank's solution electrolyte yielded an increased dissolution rate of the metallic ions, thereby leading to increase in the oxygen diffusion coefficient of the passive oxide layer formed on the titanium alloy. The morphological influence on electrochemical dissolution of 3D printed and forged Ti6Al4V alloys was investigated in deicing electrolyte by Zhou et al. [146]. This research was reportedly motivated by the use of Ti alloys in the aerospace industry and their constant exposure to deicing liquids when used for the fabrication of load-bearing components. A forged aircraft part produced via AM technique in addition to SLM and EBM manufactured specimens were investigated. Furthermore, the SLM and EBM specimens were sectioned into horizontal (SLM-H, EBM-H) and vertical (SLM-V, EBM-V) planes. Results of PP from this study showed that all the specimens exhibited similar current density and rapid deposition of corrosion products. Passive film formation is observed in all the specimens, with the EBM-H specimen displaying a broader potential range. However, the worst corrosion resistance in the test electrolyte was seen in the SLM-V specimen. The PP curves for all the specimens are displayed in Figure 20 (a-e), while Table 4 shows the corrosion parameters extrapolated from the Tafel fit. It should be noted that the higher corrosion potential depicts the stability of the passive films formed on the specimens' surface. Qin et al. [147] investigated on the corrosion behaviour and mechanism of selective laser melted Ti–35Nb alloy using mixed powder (Ti–35Nb-M) and pre-alloyed powder (Ti–35Nb–P) in Hank's solution. Both samples displayed similar electrochemical behaviour. However, the Ti–35Nb–P sample oxide appeared to be more stable compared to the mixed powder sample. This was confirmed by performing the Mott-Schottky tests, it was found that the oxide film formed on Ti–35Nb–P has lower potential range of 0.5–1.5 V, which exhibits a lower vacancy flux, a lower vacancy diffusion coefficient, and a slightly lower thickness. This shows that the pre-alloyed-based alloy exhibit superior corrosion resistance compared to the mixed alloy.

**Table 4:** Corrosion parameters extrapolated from Tafel fit of potentiodynamic polarization plots for Ti6Al4V specimens.

Specimen	E_corr_ (V)	I_corr_ (μA/cm^2^)	B_c_ (mV/dec)	B_a_ (mV/dec)
Forging	-.122	0.0176	94.5	261.2
EBM-V	-1.24	0.0311	85.4	162.9
EBM-H	-1.18	0.0113	84.3	176.8
SLM-V	1.29	0.3428	88.1	223.5
SLM-H	1.23	0.0833	84.3	352.9

## 6 Challenges and prospects for titanium-based alloys

The supply of an adequate quantity of powders for 3D printing of titanium components has been a challenge in underdeveloped countries due to the rapid increase in the demand for these powders by different manufacturing industries in the developed countries. Another setback associated with Ti alloys' use for thermomechanical processes is the heterogeneous nature of 3D printed ingots [148, 149, 150]. This led to an increase in the distribution of the alloying elements between the top, base and across the diameter of the fabricated shapes. This results in the loss of other volatile elements, which reduces the overall properties of the alloy. Furthermore, the occurrence of embrittlement at reduced temperature has also been identified when a short fabrication time (1–2 h) is utilized for fabrication at low temperatures (650–700 °C). It is, however, noteworthy that research is currently ongoing on the elimination of embrittlement, which is always a surface defect.

Ti-based alloys for applications in structural engineering depend on the modification of these alloys for improved properties. This can however be achieved through proper alloying and selection of adequate 3D printing fabrication techniques. The mechanical properties of the alloys should also be improved if the alloying elements promote the formation of new microstructures. Therefore, it is important to adequately identify the required alloy element and their possible effect on the alloy while in service. The combination of 3D printing technique such as electron beam melting with hot deformation process should improve the ductility and workability of titanium-based alloys. Conclusively, promoting the usage of titanium-based alloys for biomedical and structural applications can encourage researchers to carry out more investigation on the design and fabrication of components with titanium-based alloys.

## 7 Applications of 3D printed titanium-based alloys

3D printed titanium based alloys has been used in different applications, and some of these include tool steels used as mould inserts [151, 152], cobalt-chromium alloy for dental prostheses [153], and titanium alloy as a choice material for various medical applications as a hip endoprosthesis. Furthermore, in the biomedical industry, this class of alloy has supported the development of new organs, tissues, and biomedical implants in addition to drug delivery systems [154], its flexibility has also allowed the fabrication of complex shapes by new materials such as semi-crystalline polymeric composite [155]. This fabrication technique has been used to improve the effectiveness and efficiency of medical surgery and reduce the need for further treatment, which might help a patient fit for an implant. CAD files introduced by the National Institute of Health for AM of biomedical parts are easily transferred between various researchers [156]. This has facilitated easy reproduction of required parts across the globe.

In the aerospace industry, the fabrication of parts using AM has been greatly embraced for several reasons. First, the development of high-performance alloy to near net shape using the conventional methods has not been cost and economically efficient. The introduction of AM technique for the fabrication of aircraft body parts such as fuel nozzles (GE LEAP aero engine) [41] has been of assistance in fabricating such parts with complex designs with powders being used repeatedly without any alteration in the mechanical and physical properties of fabricated parts [157].

**Figure 21:**
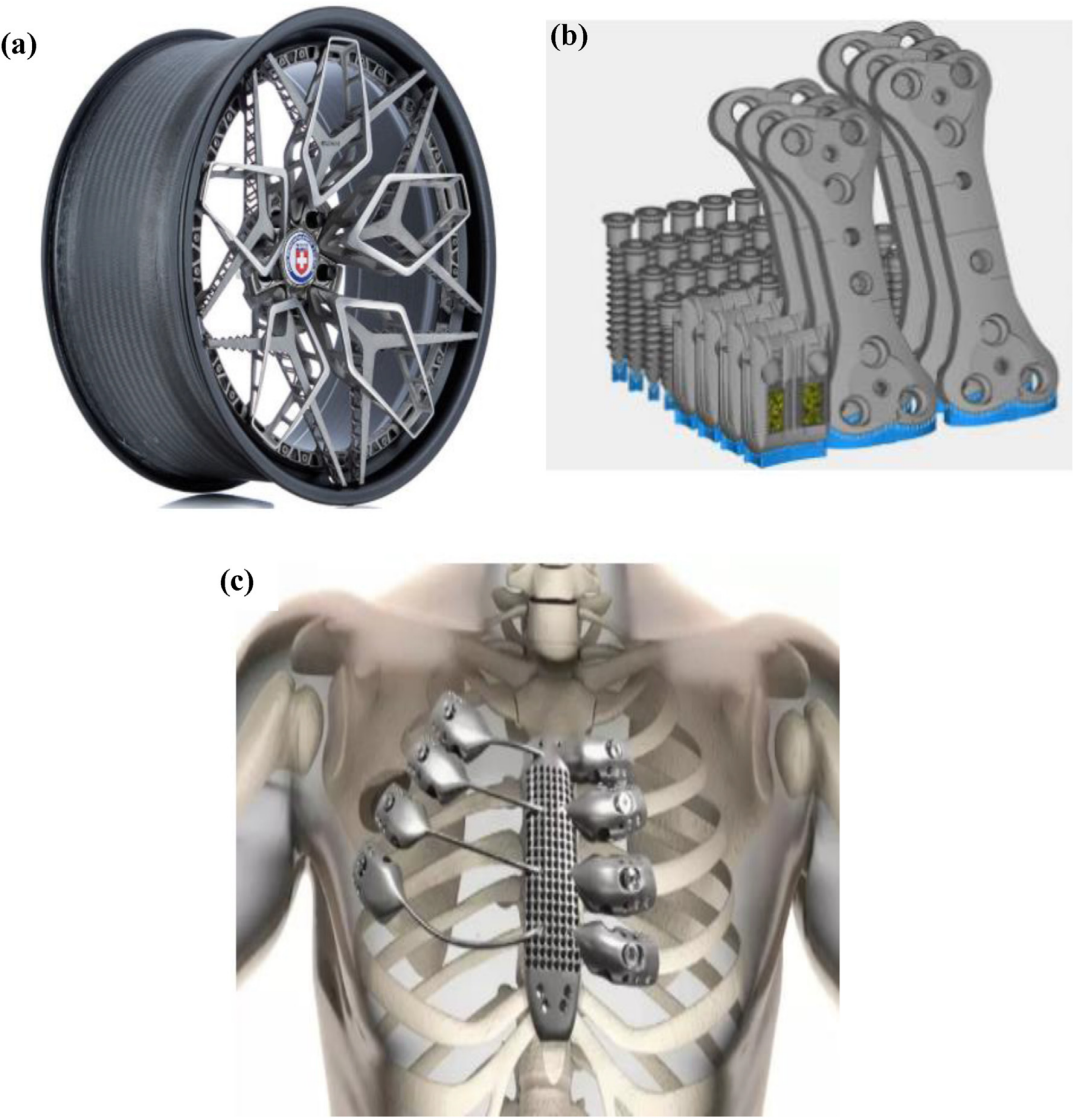
Pictorial representation of 3D printed titanium (a) car wheel [158] (b) screw, plates and cages for horse implant [159] (c) sternum implant [160].

Aside from the biomedical applications of titanium-based alloys, they are also used in the aerospace and automotive industries due to their attractive properties. These include improved resistance to oxidation, high stiffness, and low density, making them a perfect fit for lightweight applications. Pictorial representation of applications where 3D printed titanium alloy have been reportedly utilised are shown in Figure 21.

## 8 Conclusion

AM technology has shown great potential and advantages in many applications for producing complex-shaped 3D metallic components. The reviewed work showed that SLS and EBM are the most used AM techniques for fabricating metallic parts. Moreover, these techniques encounter challenges such as poor corrosion resistance because they undergo melting and solidification stages, which leads to depletion of the stable β-phase. In addition, these processes are susceptible to various metallurgical problems such as solid-state cracking due to thermal stresses from the inbuilt brittleness induced during the fabrication process. Furthermore, the fabrication of titanium based intricated shaped components for high temperature applications using the conventional techniques has proven to be difficult. However, these problems are not specific to binder jetting technique, as it operates at low temperature. It appears to be a more suitable technology that can be utilized to fabricate complex-shape titanium alloys with improved mechanical properties. Other challenges that are peculiar to 3D printed titanium-based alloys include void formation in-between material layers, which often lead to porosity during the fabrication process, thereby, decreasing the mechanical properties of the printed alloy. Although recent research has provided alternatives to some of these challenges, however, improvement is still required. This review has shown that AM of titanium-based alloys is a futuristic research area, which helps with the production of titanium-based composites with desired properties. Despite all its outstanding attributes, critical insight on production of other metallic alloys is required, with the aim of providing a deeper understanding to the relationship that exists between processing conditions and material properties.

## Declarations

### Author contribution statement

All authors listed have significantly contributed to the development and the writing of this article.

### Funding statement

This work was supported by the National Research Foundation, and University Research Committee of University of Johannesburg, South Africa.

### Data availability statement

No data was used for the research described in the article.

### Declaration of interests statement

The authors declare no conflict of interest.

### Additional information

No additional information is available for this paper.
